# Supplementary material for: IMPACT study on intervening with a manualised package to achieve treatment adherence in people with tuberculosis: protocol paper for a mixed-methods study, including a pilot randomised controlled trial
Source: BMJ Open. 2019 Dec 17;9(12):e032760. doi: 10.1136/bmjopen-2019-032760 (PMC6937100; doi:10.1136/bmjopen-2019-032760)
Supplement: Author's manuscript [file bmjopen-2019-032760.draft_revisions.pdf]

BMJ Open

BMJ Open is committed to open peer review. As part of this commitment we make the peer review history of every article we publish publicly available.

When an article is published we post the peer reviewers' comments and the authors' responses online. We also post the versions of the paper that were used during peer review. These are the versions that the peer review comments apply to.

The versions of the paper that follow are the versions that were submitted during the peer review process. They are not the versions of record or the final published versions. They should not be cited or distributed as the published version of this manuscript.

BMJ Open is an open access journal and the full, final, typeset and author-corrected version of record of the manuscript is available on our site with no access controls, subscription charges or pay-per-view fees (http://bmjopen.bmj.com).

If you have any questions on BMJ Open's open peer review process please email info.bmjopen@bmj.com

BMJ Open

The IMPACT Study: Intervening with a Manualised Package to AChieve treatment adherence in people with Tuberculosis: A mixed methods approach, including a pilot randomised controlled trial

Journal:	*BMJ Open*
Manuscript ID	bmjopen-2019-032760
Article Type:	Protocol
Date Submitted by the Author:	05-Jul-2019
Complete List of Authors:	Stagg, Helen R.; University of Edinburgh, Usher Institute<br>Abubakar, Ibrahim; University College London<br>Campbell, Colin; Public Health England, Tuberculosis Section, Centre for Infectious Disease Surveillance and Control, National Infection Service<br>Copas, Andrew; UCL<br>Darvell, Marcia; University College London<br>Horne, Robert; University College London<br>Kielmann, Karina; Queen Margaret University Edinburgh<br>Kunst, Heinke; Queen Mary University of London, Department of Respiratory Medicine<br>Mandelbaum, Mike; TB Alert<br>Pickett, Elisha; Royal Free London NHS Foundation Trust<br>Story, Alistair; University College Hospitals NHS Foundation Trust, Find&Treat<br>Vidal, Nicole; Queen Margaret University Edinburgh<br>Wurie, Fatima; University College London,<br>Lipman, Marc; Royal Free Campus, Respiratory Medicine
Keywords:	Tuberculosis < INFECTIOUS DISEASES, medication adherence, manuals, Protocols & guidelines < HEALTH SERVICES ADMINISTRATION & MANAGEMENT, pilot studies

SCHOLARONE™
Manuscripts

The IMPACT Study: Intervening with a Manualised Package to AChieve treatment adherence in people with Tuberculosis: A mixed methods approach, including a pilot randomised controlled trial

Author list: Stagg HR[1], Abubakar I[2], Campbell CNJ[2,3], Copas A[2], Darvell M[4], Horne R[5], Kielmann K[6], Kunst H[7], Mandelbaum M[8], Pickett E[9], Story A[10,11], Vidal N[6], Wurie F[3,12], Lipman M[4,9]

1. Usher Institute of Population Health Sciences and Informatics, The University of Edinburgh, MacKenzie House, 30 West Richmond Street, Edinburgh, EH8 9DX, UK
2. Institute for Global Health, University College London, 4th floor, Mortimer Market Centre, off Capper Street, London, WC1E 6JB, UK
3. TB Unit, National Infection Service, Public Health England, 61 Colindale Avenue, London NW9 5EQ, UK
4. UCL Respiratory, Division of Medicine, University College London, London, UK
5. Centre for Behavioural Medicine, UCL School of Pharmacy, Mezzanine Floor, BMA House, Tavistock Square, London, WC1H 9JP, UK
6. Institute for Global Health and Development, Queen Margaret University, Musselburgh, East Lothian, Edinburgh, EH21 6UU, UK
7. Blizard Institute, Barts and The London School of Medicine and Dentistry, Queen Mary University of London, London, E1 2AT, UK
8. TB Alert, Community Base, 113 Queens Road, Brighton, BN1 3XG, UK
9. Department of Respiratory Medicine, Royal Free London NHS Foundation Trust, Pond Street, London, NW3 2QG, UK
10. Collaborative Centre for Inclusion Health, University College London, London, UK
11. Find & Treat, University College London Hospitals NHS Foundation Trust, 235 Euston Road, London, NW1 2BU, UK
12. Department of Epidemiology and Public Health, Institute of Epidemiology and Health Care, University College London, UK

Corresponding author: Marc Lipman, Department of Respiratory Medicine, Royal Free London NHS Foundation Trust, Pond Street, London, NW3 2QG, UK; marclipman@nhs.net

Word Count: 3,900

Key words: tuberculosis, medication adherence, manuals, protocol, pilot studies

For peer review only

Abstract

Introduction

Compared to the rest of the UK and Western Europe, England has high rates of the infectious disease tuberculosis (TB). TB is curable, although treatment is for at least six months - and longer when disease is drug resistant. If patients miss too many doses (non-adherence), they may transmit infection for longer and the infecting bacteria may develop resistance to the standard drugs used for treatment. Non-adherence may therefore risk both their health and that of others. Within England, certain population groups are thought to be at higher risk of non-adherence, but the factors contributing to this have been insufficiently determined, as have the best interventions to promote adherence. The objective of this study is to develop a manualised package of interventions for use as part of routine care within National Health Services to address the social and cultural factors that lead to poor adherence to treatment for TB disease.

Methods and analysis

This study uses a mixed methods approach, with six study components. These are: (i) scoping reviews of the literature, (ii) qualitative research with patients, carers and healthcare professionals, (iii) development of the intervention, (iv) a pilot randomised controlled trial of the manualised intervention, (v) a process evaluation to examine clinical utility, and (vi) a cost analysis.

Ethics and dissemination

This study received ethics approval on 24th December 2018 from Camberwell St. Giles Ethics Committee, UK (REC reference 18/LO/1818). Findings will be published and disseminated through peer-reviewed publications and conference presentations, published in an end of study report to our funder (the National Institute for Health Research, UK), and presented to key stakeholders.

Trial Registration Number

ISRCTN 95243114

For peer review only

For peer review only - http://bmjopen.bmj.com/site/about/guidelines.xhtml

Article Summary: Strengths and limitations of this study

Strengths

- Patient-centred, mixed methods approach, based on a robust understanding of the evidence on social, cultural and personal factors that influence adherence to medication to treat tuberculosis.
- Evidence and experience of adherence captured from a variety of perspectives from across the UK, including patients, their carers, and healthcare workers.
- Generalisable patient population of individuals at risk of non-adherence across low tuberculosis (TB) incidence settings.
- Development of a pragmatic and easy-to-use tool that captures the best evidence on adherence and allows its application in the clinic setting.

Limitations

- The study culminates in a pilot trial of the manualised intervention; a larger subsequent definitive trial is needed to test whether the intervention is efficacious and cost-effective beyond any initial conclusions regarding validity and feasibility derived from the pilot.

For peer review only

For peer review only - http://bmjopen.bmj.com/site/about/guidelines.xhtml

Introduction

Against a background of rising tuberculosis (TB) in the 1990s and 2000s, the need for a comprehensive approach to TB control in England was deemed necessary by Public Health England (PHE) and the National Health Service (NHS) England. In January 2015, these bodies jointly launched the 'Collaborative Tuberculosis Strategy for England 2015-2020'[1]. This seeks to reduce TB incidence, decrease health inequalities, and ultimately contribute to international efforts to eliminate TB as a public health problem. Ensuring that people can take all of their medication as prescribed is one of the strategy's priorities, as poor adherence to treatment for TB is a driver of worse patient outcomes[2–9], increases the risk of transmission (due to delayed sputum culture conversion)[10], and can promote the development of drug resistance[3,11–16]. Subsequent National Institute for Health and Care Excellence (NICE) guidance noted the lack of robust TB research in this area[17].

Barriers to optimal adherence to treatment for TB may occur for a number of reasons. These include:

- patient-related factors, including perceptions and beliefs,
- cultural influences and current mental state,
- structural economic factors and social support networks,
- health service factors that include treatment complexity as well as accessibility of those services and the relationships patients develop with service providers[18,19].

Non-adherence is not a single issue and may take various forms e.g. suboptimal implementation (skipping doses), or stopping treatment early (for example, as soon as a patient feels better; this is discontinuation)[20].

Although a series of studies have been undertaken to define the population groups most at risk of non-adherence[19], it is currently difficult, prior to starting medication, to identify who may struggle with taking treatment as prescribed. To date, methods to support treatment address some, but not all, of the important underlying reasons for poor adherence. For example, the World Health Organization's recent focus on digital technologies reflects our attention on individual-level determinants of adherence and reminder/observation-based systems[21]; far less research has addressed the social and structural barriers to staying on TB treatment.

In the UK, the development of an intervention to support adherence to treatment that is sensitive to the individual's cultural background and social circumstances, and can be routinely delivered within the NHS, is critical. To this purpose, a mixed-methods, patient-centred, approach to the study of the modifiable factors that influence patients' adherence to treatment for TB is required.

Methods and analysis

Research question

Can a manualised intervention be developed to address the social, cultural, and structural barriers that lead to non-adherence to treatment in NHS patients with active TB?

Aim

To develop, pilot, and evaluate process and interim outcomes for an effective manualised intervention that improves the likelihood of treatment completion among NHS patients at risk of poor adherence due to social, cultural, and structural factors.

For peer review only - http://bmjopen.bmj.com/site/about/guidelines.xhtml

Objectives

1) Synthesise current knowledge on (a) determinants of adherence to treatment for TB, and (b) interventions that can support adherence, with particular emphasis on social and cultural barriers. (Scoping Review and Conceptual Framework)
2) Apply a conceptual framework of adherence endorsed by NICE guidelines (the Perceptions and Practicalities (PAPA) approach [18] to elucidate and address the personal, socio-cultural, and health systems context, mechanisms, and pathways of poor adherence among NHS patients with TB. (Formative Research)
3) Develop a manualised intervention with multiple components that can identify (a) NHS patients most at risk of non-adherence, (b) the salient modifiable barriers, and (c) the tailored support mechanisms required to meet individual patient needs by matching appropriate interventions to specific barriers, as recommended by NICE. (Development of Intervention)
4) Pilot the intervention package in people at risk of poor adherence to define how the components work in combination and separately. (Pilot Study)
5) Evaluate the process of implementation of this intervention through describing the challenges and facilitators in delivering the package as intended (fidelity, reach) and assessing the impact of the intervention through evaluation of adherence indicators. (Process Evaluation)
6) Use the findings of the pilot to assess the costs of delivering the manualised intervention in an NHS setting, and to guide development of a proposal for a full randomised controlled trial (RCT).

Study design

This study uses a mixed methods approach. There are six sub-sections, reflecting the six objectives: i) Scoping Review and Conceptual Framework, ii) Formative Research, iii) Development of Intervention, iv) Pilot Study, v) Process Evaluation, vi) Cost Analysis and Future Work. Although the different elements are described separately below, the research activities for each will overlap, and some will run concurrently. The full programme of work and relationships between subsections is shown in Figure 1.

i. Scoping Review

To underpin the development of the manualised intervention, the first study sub-section will undertake literature reviews to answer the following research questions:

i) What personal, social, cultural, health systems-related and structural factors affect individuals' ability to adhere to treatment for TB?
ii) What kinds of intervention have been developed to address the multiple levels (personal, social, cultural, systems and structural) at which barriers to adherence may operate?
iii) What is the evidence for the successful impact of interventions to address barriers to adherence to treatment for TB?

These questions will be answered through the three following reviews:

1) A scoping review of qualitative studies that examine the personal, social, cultural, health systems-related and structural factors affecting adherence to treatment for TB from the perspectives of adult patients, care givers, or health care providers, as well as studies that evaluate interventions to support adherence to treatment for TB.

For peer review only - http://bmjopen.bmj.com/site/about/guidelines.xhtml

2) A critical review of quantitative studies examining the personal, social, cultural, health systems-related and structural factors affecting adherence to treatment for TB.

3) A critical review of quantitative studies that have examined the effectiveness of interventions to improve adherence in people taking treatment for TB, building on existing systematic reviews, including the provision and delivery of information and/or education; enablers and/or incentives; social support; case management approaches.

Together, these three reviews will enable us to conduct a critical interpretive synthesis of findings from qualitative and quantitative studies examining the assumptions and mechanisms of effect underlying interventions to improve adherence to treatment for TB.

ii. Formative Research

Formative research methods, recruitment and eligibility

In line with findings from the scoping review, we will develop topic guides for qualitative interviews exploring current models of TB service delivery in the study sites, providers' views on the barriers and facilitators for treatment adherence, and TB patients' and their family members' experiences of starting and staying on treatment. The thematic analysis of these interviews will complement insights gained from the scoping reviews which will allow us to refine a conceptual framework of adherence building on the Perceptions and Practicalities Approach (PAPA) [18]. Approximately 60 participants will be enrolled.

Adults (aged 18 and over) who are currently taking or recently completed treatment will be identified by the local TB service at four UK sites (Edinburgh, London [Central London & East London] and Southampton) and asked to take part in the study. The services have been chosen for their patient diversity, geographic spread and as reflecting the national TB picture. The patient group will be enriched with people who have been poorly adherent to treatment, although we will also include patients who report full adherence so that we can capture what may have enabled them to take treatment as prescribed. With patient consent, family members and/or carers will also be approached and asked to participate.

Health and social care workers from both primary and secondary care settings will be directly approached by researchers and invited to be interviewed. All those approached will be aged 18 or over and involved in TB management and care.

Data collection methods

Three different methods of data collection will be used to undertake the formative research.

1) **In-depth interviews – patients and family members/carers**

These will be conducted as individual interviews with approximately 30 participants, using a topic guide. The areas to be explored are: self-perception; personal beliefs and practices related to medicine-taking; health literacy and health-seeking behaviour; social support; cultural norms around health-seeking behaviour; financial and other structural barriers.

For peer review only - http://bmjopen.bmj.com/site/about/guidelines.xhtml

2) Cognitive assessments - patients

To ensure that the validated questionnaires (i.e. Beliefs about Medicines Questionnaires[22], and the Brief Illness Perception Questionnaire[23], are accessible and acceptable to patients, we will also conduct cognitive interviews with 10 patients. We will then have confidence to use them in the later pilot study to assess patient perceptions and practicalities affecting adherence to anti-TB therapy. These will explore self-perception; personal beliefs; and practices related to medicine-taking.

3) Semi-structured Interviews – healthcare providers

These will be undertaken with health care providers responsible for multiple aspects of TB care (doctors, nurses, social workers, directly observed therapy [DOT] providers, managers and administrators). They will focus on providers' perceptions of factors affecting patient understanding of TB and its treatment; service delivery models including staffing; organisation of care; communication. We will interview four to six providers at each site, aiming for a total of 20 interviews.

iii. Development of the manualised intervention

Intervention development process

The data collected from the scoping reviews and formative research will be presented to, and synthesised by, an Intervention Development Group (IDG). A manualised intervention will be developed to identify (a) NHS patients most at risk of non-adherence, (b) their salient modifiable barriers, and (c) the tailored support mechanisms that meet individual patient needs by matching appropriate interventions to these specific barriers.

The IDG will include:

- patients (in particular the homeless, ethnic minorities, migrants new to the UK, and patients with drug resistant TB)
- family members/significant others of affected persons
- members of the public
- health care professionals (from both primary and secondary care)
- other professionals who work with patients/communities affected by TB

The constitution of the IDG will enable a co-production approach to ensure that the intervention is pragmatic, can be delivered within the context of existing care pathways, and is of benefit to service users and those who are likely to access the intervention.

Intervention contents

The manualised intervention is likely to consist of a screening and assessment tool, in addition to a package of measures that can be tailored to the needs of individual patients from different population groups.

As patterns of adherence may be irregular over time, it is intended that the manualised intervention will be administered to all patients at each patient review. The tool thus needs to be quick and easy to administer. It may be electronically linked to patient records, enabling a comprehensive picture of the risk of possible non-adherence to be developed for each patient, as well as within a clinic population. The various delivery options for the intervention (such as using paper or an app) will be considered during its development stage.

The menu of supportive measures may, for example, include:

For peer review only

For peer review only - http://bmjopen.bmj.com/site/about/guidelines.xhtml

- Informational intervention: e.g. providing a convincing story setting out the rationale and on-going need for medication, addressing concerns about potential adverse effects and consequences of treatment and what to do if such events occur e.g. the participant will be informed that it is possible to change their treatment regimens to alternatives[24].
- Practicalities and Capability based interventions: video observed therapy [VOT], DOT, reminders including text messaging, automated methods for monitoring and feedback including electronic dosette boxes, use of a medication app, incentives e.g. financial and food vouchers, mitigation and management of drug toxicity due to treatments.
- Social and system interventions: offering flexibility in appointments; enhanced guidance on 'navigating' clinic pathways; signposting patients to relevant services, e.g. housing, drug and alcohol services, and social care; providing peer-support.

iv. Piloting the intervention

Study design, recruitment and eligibility

Once the intervention is developed, proof-of-concept is required within the real world. This will be undertaken using a cluster randomised pilot study that compares the manualised intervention to the usual standard of care in four London clinics treating TB. Two clinics will be randomly allocated to the intervention and two to standard of care. Participants starting treatment for TB will be enrolled. These are likely to include people at greater risk of poor adherence such as migrants newly arrived in the UK, people whose first language is not English, people with a mental health disorder, people taking immunosuppressive therapy or known to have immunodeficiency, those with a previous history of treatment for TB, or poor-adherence with anti-TB medication, and people with a current or previous history of drug or alcohol misuse.

Patients included in the pilot study will be aged 18 or over and will be started on treatment for TB, irrespective of site of disease. We will exclude patients who are unable to provide informed consent, those already on treatment and those who are not expected to live for the duration of the study (a minimum of six months from starting treatment).

The purpose of the pilot study is not formal hypothesis testing. Given this, a target sample size of 80 patients enrolled (20 per site) was identified for the pilot study as providing useful information that can help determine whether the intervention will be deliverable within a clinical setting. It will also guide the development of a possible larger definitive study using the intervention. The four TB clinics of interest (in East and North London) each treat in excess of 60 relevant patients per annum.

Based on usage of DOT within the clinic populations seen at the treatment sites (i.e. individuals currently identified as needing adherence support), we estimate that around 33% of patients will be at risk of non-adherence. Taking this as a minimum (as the manualised intervention is likely to be more sensitive than current risk assessments), we would expect that at least 26 of the 80 patients recruited will be identified as requiring adherence support. This sample size allows us to measure consent to enrolment for 80 individuals, data completeness for adherence and treatment outcomes for 80 individuals, data on acceptability and feasibility of the intervention package for around 40 individuals, and data on acceptability and feasibility of adherence support for at least 26 individuals (13 receiving the manualised intervention and 13 standard care).

Outcomes of the pilot

The primary outcome of the study will be level of adherence, measured as the proportion of prescribed doses taken and assessed at six months from the start of treatment. In addition to this

For peer review only - http://bmjopen.bmj.com/site/about/guidelines.xhtml

primary outcome, a number of secondary outcomes will also be measured, as follows:

1) Proportion consenting to the study.
2) Completeness of data for measures of adherence and treatment completion.
3) Proportion of patients withdrawing during the study and the reasons why.
4) Proportion of patients identified as needing adherence support in the intervention arm.
5) Proportion of patients offered adherence support and accepting it in the intervention arm.
6) Documentation of which adherence-promoting activities have been implemented among patients both in the standard of care and intervention arm, and when.
7) Detailed treatment implementation information: e.g. proportion of patients completing treatment, proportion of patients still on treatment after nine months or at study completion (whichever is the earlier).
8) Patterns of adherence (implementation and discontinuation).
9) Impact of manualised intervention on maintaining adherence over the duration of treatment.
10) Process variables – adherence-related perceptions and practicalities.

Measures of treatment completion and adherence

Our primary measure of adherence will be pill counts performed by the research nurse or clinical team. Other measures will also be used, and compared with pill counts. Patients within the study will be asked to bring their medication to each appointment, so that it can be counted and compared to expected levels based on what has been prescribed. In the case of DOT or VOT methods being used, a record of missed doses will be kept.

At each appointment, participants will also be asked to provide a 5ml sample of urine to check for adherence to the prescribed anti-TB medication; and they will be asked directly whether they have managed to take all their medicines and if they have missed any doses [25].

Administration of the manualised intervention

The patient's case manager (usually the TB clinic nurse), plus a study research nurse, will apply the intervention in partnership with the patient to identify whether personal, socio-cultural and/or systems risk factors are present that suggest likely poor adherence with treatment. If these are identified, then the relevant measures outlined in the manualised intervention that may mitigate these will be reviewed and implemented with the agreement of the participant. These will be continued throughout the course of treatment, or stopped if no longer deemed to be relevant or required on reassessment.

Study schedule of visits
Study subjects will be seen at weeks 0, 2, 4, 8, 12, 16, 20 and 24 (earliest date of treatment completion). Should they require on-going treatment after six months, they will be seen as clinically indicated. At each review, adherence assessments will be performed in addition to an assessment of perceptions and practicalities, the completion of the BMQ and the EQ-5D-5L Quality of Life questionnaire[22,26]. The manualised intervention will be applied if the patient is attending a clinic that has been randomised to the intervention arm.

Follow Up
Most patients who do not have clinically important drug resistant disease receive six months of treatment. To allow for treatment interruptions, patients within the pilot study will be followed to either treatment completion or for a total of nine months from starting anti-TB therapy.

For peer review only - http://bmjopen.bmj.com/site/about/guidelines.xhtml

For peer review only

Analysis and interpretation
Where possible, univariable analyses such as Fisher's exact test will be undertaken to compare each outcome measure listed earlier between study arms (intervention and control). An assessment of the balance in baseline characteristics between the study arms will also be conducted. If randomisation has failed to evenly distribute key baseline characteristics (e.g. age, sex, ethnicity, or other factors identified as important during the scoping reviews), then multivariable logistic regression analyses adjusting for these factors will also be undertaken, subject to constraints on the degree of adjustment, which depend on the prevalence of the outcome and sample size. Inter-site variability in outcomes will be measured. The need to adjust for clustering by clinical care provider will be assessed; clustering by site alone cannot be formally adjusted for due to the modest number of sites in the pilot study.

The analysis of the first three of our secondary outcomes will address the feasibility of a definitive trial following a similar design to the pilot. Analysis of secondary outcomes four to six addresses the intervention, and complements the process evaluation (see below). Analysis of the primary outcome and final secondary outcomes around treatment adherence and completion provides initial information - given the modest sample size - concerning the effectiveness of the intervention, and may assist the sample size calculation for the definitive trial. They can also offer an alert in the unlikely event that the intervention is harmful.

v. Process evaluation

Evaluation method
We will evaluate the implementation process by analysing the challenges and facilitators in delivering the package. The impact of the intervention will be assessed by evaluation of adherence indicators. We will use the findings of the pilot to assess the costs of delivering the manualised intervention in an NHS setting, and to guide development of a proposal for a full RCT.

The process evaluation will consist of a description of the process of intervention implementation. It will assess how well the manualised intervention achieves its intended aim compared to standard care.

We will consider:
1) The fidelity of the intervention as delivered in comparison to how it was designed and envisaged,
2) The reach of the intervention (the proportion of the target group receiving it),
3) The barriers to facilitating implementation of the intervention and how these can be addressed,
4) The pre-existing factors that facilitated implementation.

Process measures, recruitment and eligibility
Process measures for each element of the package will be developed once the manual development has been completed, and used to assess success. They will include acceptability, uptake and change in practice. We will work with patients and staff separately at all four London sites. We will interview 20 patients (five at each study site i.e. 10 from each arm); and, if possible, 20 health care workers (five at each site). The patients will be selected within each site using purposeful sampling of clinic lists of every fourth patient with active TB.

Key outputs will include a qualitative evaluation of delivery, the development of a narrative description of the process of intervention implementation and maintenance and a quantitative

For peer review only - http://bmjopen.bmj.com/site/about/guidelines.xhtml

assessment of adherence-related perceptions and practicalities within intervention and control groups.

We will invite participation in the process evaluation from patients enrolled in the pilot study or staff members treating patients at one of the four London sites also involved in delivering the pilot study. They will be included if they are aged 18 or over and able to provide informed consent.

vi. Cost Analysis and Future Work

In order to generate realistic estimates of the cost of the intervention, cost data from the NHS perspective will be collected during the pilot study using NICE guideline implementation tools.

After the pilot study and process evaluation have been completed, a final intervention package will be designed for use in a definitive RCT of the manualised package of interventions. The design of this final package will be based on the results of the process evaluation and the experience gained during the piloting of the intervention, modifying the definitive trial design and/or data collection accordingly.

Patient and public engagement

As documented above, patient representatives will sit on the IDG for the study. In addition, TB Alert, the UK's only national TB charity, has membership of the IDG. The role of the IDG, which will meet regularly throughout the study, is described in section iii. At the end of the study, the IDG will be involved in commenting on the findings and contributing to the dissemination plan.

Ethics and dissemination

This study received ethics approval on 24th December 2018 from Camberwell St Giles Ethics Committee (REC reference 18/LO/1818). Findings will be published and disseminated through peer-reviewed publications and conference presentations, published in an end of study report to our funder (the National Institute for Health Research, UK), and presented to key stakeholders.

Conclusion

Our study will develop and pilot a manualised intervention to improve adherence to treatment for TB in the UK using a mixed methods, patient-centred and provider-informed approach. This will enable us to begin to understand what motivates patients' treatment behaviour, whilst ensuring deliverability within the NHS. Our work is based on a robust understanding of the evidence on social, cultural and personal factors that influence adherence, and the interventions that are most effective in addressing these. The study reflects the geographic spread of TB in the UK and captures not only patient and expert clinical and academic experience, but also that of family and carers to develop the intervention. A key feature of the study is the co-production of a pragmatic and easy-to-use tool that utilises the best evidence on adherence, and allows its application in the clinic setting in a dynamic and iterative way.

Although the final pilot study may be limited to a relatively small sample size, it is hoped that its broad patient-centred perspective will make a useful contribution to our understanding of, and ability to deal effectively with, the risks of non-adherence to TB treatment in a population that can find this challenging. As many of the factors influencing adherence are likely to be generalisable to patients with other conditions in both high and low resource settings, this study also has the potential to inform adherence interventions in other disease areas.

For peer review only - http://bmjopen.bmj.com/site/about/guidelines.xhtml

Authors' contributions

ML, IA, RH, KK and HRS conceived of the work. HRS, IA, AC, RH, KK, MM, AS, NV and ML designed the work. HRS, CNJC, MD, RH, KK, drafted the manuscript. All authors critically revised the manuscript and gave final approval of the version of the protocol manuscript to be published. All authors agree to be accountable for all aspects of the work in ensuring that questions related to the accuracy or integrity of any part are appropriately investigated and resolved.

Funding statement

This work was supported by the National Institute for Health Research (NIHR) Health Technology Assessment Programme, UK grant number 16/88/06.

The views expressed are those of the author(s) and not necessarily those of the National Health Service, UK, the NIHR or the Department of Health and Social Care.

Competing interests statement

IA, MD, HK, KK, ML, MM, EP, AS, FW have no competing interests to declare.

RH is supported by the National Institute for Health Research (NIHR, Collaboration for Leadership in Applied Health Research and Care (CLAHRC), North Thames at Bart's Health NHS Trust and Asthma UK (AUKCAR). Speaker engagements with honoraria with the following companies: Abbvie, Amgen, Astellas, AstraZeneca, Biogen, Erasmus, Idec, Gilead Sciences, GlaxoSmithKline, Janssen, Merck Sharp Dohme, Novartis, Pfizer, Roche, Shire Pharmaceuticals and TEVAProfessor Rob Horne is Founding Director of a UCL-Business spin-out company (Spoonful of Sugar Ltd) providing consultancy on treatment engagement and patient support programmes to healthcare policy makers, providers and industry.

HRS reports grants from Medical Research Council (MRC), UK, grants from National Institute for Health Research (NIHR), UK, during the conduct of the study; other from Korean CDC and Johnson and Johnson (makers of Bedaquiline), other from Latvian Society Against Tuberculosis (funding through Otsuka and Johnson and Johnson), outside the submitted work.

CNJC reports personal fees from Public Health England, outside the submitted work.

For peer review only

References

1 Public Health England. Collaborative Tuberculosis Strategy for England 2015 to 2020. London: 2015. https://www.gov.uk/government/publications/collaborative-tuberculosis-strategy-for-england

2 Imperial MZ, Nahid P, Phillips PPJ, *et al.* A patient-level pooled analysis of treatment-shortening regimens for drug-susceptible pulmonary tuberculosis. *Nat Med* 2018;**24**:1708–15. doi:10.1038/s41591-018-0224-2

3 Kayigamba FR, Bakker MI, Mugisha V, *et al.* Adherence to Tuberculosis Treatment, Sputum Smear Conversion and Mortality: A Retrospective Cohort Study in 48 Rwandan Clinics. *PLoS One* 2013;**8**:e73501. doi:10.1371/journal.pone.0073501

4 Thomas A, Gopi PG, Santha T, *et al.* Predictors of relapse among pulmonary tuberculosis patients treated in a DOTS programme in South India. *Int J Tuberc Lung Dis* 2005;**9**:556–61.http://www.ncbi.nlm.nih.gov/pubmed/15875929 (accessed 1 May 2019).

5 Valencia S, León M, Losada I, *et al.* How do we measure adherence to anti-tuberculosis treatment? *Expert Rev Anti Infect Ther* 2017;**15**:157–65. doi:10.1080/14787210.2017.1264270

6 van den Boogaard J, Boeree MJ, Kibiki GS, *et al.* The complexity of the adherence-response relationship in tuberculosis treatment: why are we still in the dark and how can we get out? *Trop Med Int Health* 2011;**16**:693–8. doi:10.1111/j.1365-3156.2011.02755.x

7 Zhdanov V, Bilenko N, Mor Z. Risk Factors for Recurrent Tuberculosis among Successfully Treated Patients in Israel, 1999-2011. *Isr Med Assoc J* 2017;**19**:237–41.http://www.ncbi.nlm.nih.gov/pubmed/28480678 (accessed 1 May 2019).

8 Bastard M, Sanchez-Padilla E, Hewison C, *et al.* Effects of treatment interruption patterns on treatment success among patients with multidrug-resistant tuberculosis in Armenia and Abkhazia. *J Infect Dis* 2015;**211**:1607–15. doi:10.1093/infdis/jiu551

9 Podewils LJ, Gler MTS, Quelapio MI, *et al.* Patterns of Treatment Interruption among Patients with Multidrug-Resistant TB (MDR TB) and Association with Interim and Final Treatment Outcomes. *PLoS One* 2013;**8**:e70064. doi:10.1371/journal.pone.0070064

10 Chirwa T, Nyasulu P, Chirwa E, *et al.* Levels of tuberculosis treatment adherence among sputum smear positive pulmonary tuberculosis patients attending care at Zomba Central hospital, southern Malawi, 2007-2008. *PLoS One* 2013;**8**:e63050. doi:10.1371/journal.pone.0063050

11 Arnold A, Cooke GS, Kon OM, *et al.* Drug resistant TB: UK multicentre study (DRUMS): Treatment, management and outcomes in London and West Midlands 2008–2014. *J Infect* 2017;**74**:260–71. doi:10.1016/j.jinf.2016.12.005

12 Pritchard AJ, Hayward AC, Monk PN, *et al.* Risk factors for drug resistant tuberculosis in Leicestershire--poor adherence to treatment remains an important cause of resistance. *Epidemiol Infect* 2003;**130**:481–3.http://www.ncbi.nlm.nih.gov/pubmed/12825732 (accessed 30 Jan 2019).

13 Cadosch D, Abel Zur Wiesch P, Kouyos R, *et al.* The Role of Adherence and Retreatment in De Novo Emergence of MDR-TB. *PLoS Comput Biol* 2016;**12**:e1004749. doi:10.1371/journal.pcbi.1004749

14 Saunders NJ, Trivedi UH, Thomson ML, *et al.* Deep resequencing of serial sputum isolates of Mycobacterium tuberculosis during therapeutic failure due to poor compliance reveals stepwise mutation of key resistance genes on an otherwise stable genetic background. *J Infect* 2011;**62**:212–7. doi:10.1016/j.jinf.2011.01.003

15 Shin SS, Keshavjee S, Gelmanova IY, *et al.* Development of Extensively Drug-resistant Tuberculosis during Multidrug-resistant Tuberculosis Treatment. *Am J Respir Crit Care Med* 2010;**182**:426–32. doi:10.1164/rccm.200911-1768OC

16 Bradford WZ, Martin JN, Reingold AL, *et al.* The changing epidemiology of acquired drug-resistant tuberculosis in San Francisco, USA. *Lancet* 1996;**348**:928–31. doi:10.1016/S0140-6736(96)03027-9

For peer review only - http://bmjopen.bmj.com/site/about/guidelines.xhtml

17 NICE. NICE Guideline: Tuberculosis. Natl. Inst. Heal. Care Excell. 2016;:178.
18 Horne R, Weinman J, Barber N, *et al.* Concordance, adherence and compliance in medicine taking. 2005. doi:10.1007/SpringerReference_64584
19 De Geest S, Sabaté E. Adherence to long-term therapies: Evidence for action. World Health Organization. Eur. J. Cardiovasc. Nurs. 2003. doi:10.1016/S1474-5151(03)00091-4
20 Vrijens B, De Geest S, Hughes DA, *et al.* A new taxonomy for describing and defining adherence to medications. *Br J Clin Pharmacol* 2012;**73**:691–705. doi:10.1111/j.1365-2125.2012.04167.x
21 World Health Organisation. *Handbook for the use of digital technologies to support tuberculosis medication adherence*. Geneva, Switzerland: 2017.
22 Horne R, Weinman J, Hankins M. The beliefs about medicines questionnaire: The development and evaluation of a new method for assessing the cognitive representation of medication. *Psychol Heal* 1999;**14**:1–24. doi:10.1080/08870449908407311
23 Broadbent E, Petrie KJ, Main J, *et al.* The Brief Illness Perception Questionnaire. *J Psychosom Res* 2006;**60**:631–7. doi:10.1016/j.jpsychores.2005.10.020
24 Michie S, van Stralen MM, West R. The behaviour change wheel: A new method for characterising and designing behaviour change interventions. *Implement Sci* 2011;**6**. doi:10.1186/1748-5908-6-42
25 Weiss JJ, Bräu N, Stivala A, *et al.* Review article: adherence to medication for chronic hepatitis C - building on the model of human immunodeficiency virus antiretroviral adherence research. *Aliment Pharmacol Ther* 2009;**30**:14–27. doi:10.1111/j.1365-2036.2009.04004.x
26 Herdman M, Gudex C, Lloyd A, *et al.* Development and preliminary testing of the new five-level version of EQ-5D (EQ-5D-5L). *Qual Life Res* 2011;**20**:1727–36. doi:10.1007/s11136-011-9903-x

For peer review only

Figure legends

Figure 1- The IMPACT study
The six mixed-methods elements of the IMPACT [Intervening with a Manualised Package to Achieve treatment adherence in people with Tuberculosis] study and the relationships between them. The study is guided by a steering group, which has oversight over the entire process and culminates in a pilot study with associated evaluatory elements.

For peer review only

For peer review only - http://bmjopen.bmj.com/site/about/guidelines.xhtml

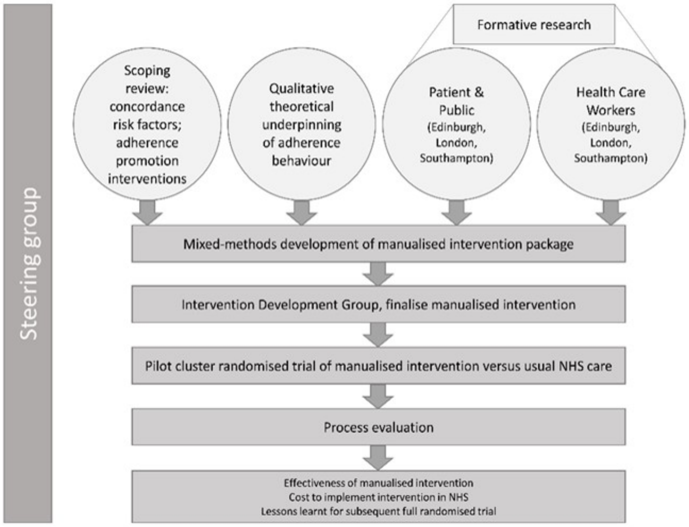

Figure 1- The IMPACT study
The six mixed-methods elements of the IMPACT [Intervening with a Manualised Package to Achieve treatment adherence in people with Tuberculosis] study and the relationships between them. The study is guided by a steering group, which has oversight over the entire process and culminates in a pilot study with associated evaluatory elements.

159x92mm (150 x 150 DPI)

BMJ Open

The IMPACT Study: Intervening with a Manualised Package to AChieve treatment adherence in people with Tuberculosis: A mixed methods approach, including a pilot randomised controlled trial

Journal:	*BMJ Open*
Manuscript ID	bmjopen-2019-032760.R1
Article Type:	Protocol
Date Submitted by the Author:	18-Sep-2019

Complete List of Authors:	Stagg, Helen R.; University of Edinburgh, Usher Institute<br>Abubakar, Ibrahim; University College London<br>Campbell, Colin; Public Health England, Tuberculosis Section, Centre for Infectious Disease Surveillance and Control, National Infection Service<br>Copas, Andrew; UCL<br>Darvell, Marcia; University College London<br>Horne, Robert; University College London<br>Kielmann, Karina; Queen Margaret University Edinburgh<br>Kunst, Heinke; Queen Mary University of London, Department of Respiratory Medicine<br>Mandelbaum, Mike; TB Alert<br>Pickett, Elisha; Royal Free London NHS Foundation Trust<br>Story, Alistair; University College Hospitals NHS Foundation Trust, Find&Treat<br>Vidal, Nicole; Queen Margaret University Edinburgh<br>Wurie, Fatima; University College London,<br>Lipman, Marc; Royal Free Campus, Respiratory Medicine

**Primary Subject Heading**:	Respiratory medicine
Secondary Subject Heading:	Patient-centred medicine, Infectious diseases, Epidemiology, Qualitative research
Keywords:	Tuberculosis < INFECTIOUS DISEASES, medication adherence, manuals, Protocols & guidelines < HEALTH SERVICES ADMINISTRATION & MANAGEMENT, pilot studies

SCHOLARONE™
Manuscripts

The IMPACT Study: Intervening with a Manualised Package to AChieve treatment adherence in people with Tuberculosis: A mixed methods approach, including a pilot randomised controlled trial

Author list: Stagg HR[1], Abubakar I[2], Campbell CNJ[2,3], Copas A[2], Darvell M[4], Horne R[5], Kielmann K[6], Kunst H[7], Mandelbaum M[8], Pickett E[9], Story A[10,11], Vidal N[6], Wurie F[3,12], Lipman M[4,9]

1. Usher Institute of Population Health Sciences and Informatics, The University of Edinburgh, MacKenzie House, 30 West Richmond Street, Edinburgh, EH8 9DX, UK
2. Institute for Global Health, University College London, 4th floor, Mortimer Market Centre, off Capper Street, London, WC1E 6JB, UK
3. TB Unit, National Infection Service, Public Health England, 61 Colindale Avenue, London NW9 5EQ, UK
4. UCL Respiratory, Division of Medicine, University College London, London, UK
5. Centre for Behavioural Medicine, UCL School of Pharmacy, Mezzanine Floor, BMA House, Tavistock Square, London, WC1H 9JP, UK
6. Institute for Global Health and Development, Queen Margaret University, Musselburgh, East Lothian, Edinburgh, EH21 6UU, UK
7. Blizard Institute, Barts and The London School of Medicine and Dentistry, Queen Mary University of London, London, E1 2AT, UK
8. TB Alert, Community Base, 113 Queens Road, Brighton, BN1 3XG, UK
9. Department of Respiratory Medicine, Royal Free London NHS Foundation Trust, Pond Street, London, NW3 2QG, UK
10. Collaborative Centre for Inclusion Health, University College London, London, UK
11. Find & Treat, University College London Hospitals NHS Foundation Trust, 235 Euston Road, London, NW1 2BU, UK
12. Department of Epidemiology and Public Health, Institute of Epidemiology and Health Care, University College London, UK

Corresponding author: Marc Lipman, Department of Respiratory Medicine, Royal Free London NHS Foundation Trust, Pond Street, London, NW3 2QG, UK; marclipman@nhs.net

Word Count: 4,327

Key words: tuberculosis, medication adherence, manuals, protocol, pilot studies

w only

For peer review only - http://bmjopen.bmj.com/site/about/guidelines.xhtml

Abstract

Introduction

Compared to the rest of the UK and Western Europe, England has high rates of the infectious disease tuberculosis (TB). TB is curable, although treatment is for at least six months - and longer when disease is drug resistant. If patients miss too many doses (non-adherence), they may transmit infection for longer and the infecting bacteria may develop resistance to the standard drugs used for treatment. Non-adherence may therefore risk both their health and that of others. Within England, certain population groups are thought to be at higher risk of non-adherence, but the factors contributing to this have been insufficiently determined, as have the best interventions to promote adherence. The objective of this study is to develop a manualised package of interventions for use as part of routine care within National Health Services to address the social and cultural factors that lead to poor adherence to treatment for TB disease.

Methods and analysis

This study uses a mixed methods approach, with six study components. These are: (i) scoping reviews of the literature, (ii) qualitative research with patients, carers and healthcare professionals, (iii) development of the intervention, (iv) a pilot randomised controlled trial of the manualised intervention, (v) a process evaluation to examine clinical utility, and (vi) a cost analysis.

Ethics and dissemination

This study received ethics approval on 24th December 2018 from Camberwell St. Giles Ethics Committee, UK (REC reference 18/LO/1818). Findings will be published and disseminated through peer-reviewed publications and conference presentations, published in an end of study report to our funder (the National Institute for Health Research, UK), and presented to key stakeholders.

Trial Registration Number

ISRCTN 95243114

For peer review only

Article Summary: Strengths and limitations of this study

Strengths

- Patient-centred, mixed methods approach, based on a robust understanding of the evidence on social, cultural and personal factors that influence adherence to medication to treat tuberculosis.
- Evidence and experience of adherence captured from a variety of perspectives from across the UK, including patients, their carers, and healthcare workers.
- Generalisable patient population of individuals at risk of non-adherence across low tuberculosis (TB) incidence settings.
- Development of a pragmatic and easy-to-use tool that captures the best evidence on adherence and allows its application in the clinic setting.

Limitations

- The study culminates in a pilot trial of the manualised intervention; a larger subsequent definitive trial is needed to test whether the intervention is efficacious and cost-effective beyond any initial conclusions regarding validity and feasibility derived from the pilot.

For peer review only

For peer review only - http://bmjopen.bmj.com/site/about/guidelines.xhtml

Introduction

Against a background of rising tuberculosis (TB) in the 1990s and 2000s, the need for a comprehensive approach to TB control in England was deemed necessary by Public Health England (PHE) and the National Health Service (NHS) England. In January 2015, these bodies jointly launched the 'Collaborative Tuberculosis Strategy for England 2015-2020'[1]. This seeks to reduce TB incidence, decrease health inequalities, and ultimately contribute to international efforts to eliminate TB as a public health problem. Ensuring that people can take all of their medication as prescribed is one of the strategy's priorities, as poor adherence to treatment for TB is a driver of worse patient outcomes[2–9], increases the risk of transmission (due to delayed sputum culture conversion)[10], and can promote the development of drug resistance[3,11–16]. Subsequent National Institute for Health and Care Excellence (NICE) guidance noted the lack of robust TB research in this area[17].

Barriers to optimal adherence to treatment for TB may occur for a number of reasons. These include:

- patient-related factors, including perceptions and beliefs,
- cultural influences and current mental state,
- structural economic factors and social support networks,
- health service factors that include treatment complexity as well as accessibility of those services and the relationships patients develop with service providers[18,19].

Non-adherence is not a single issue and may take various forms e.g. suboptimal implementation (skipping doses), or stopping treatment early (for example, as soon as a patient feels better; this is discontinuation)[20].

Although a series of studies have been undertaken to define the population groups most at risk of non-adherence[19], it is currently difficult, prior to starting medication, to identify who may struggle with taking treatment as prescribed. To date, methods to support treatment address some, but not all, of the important underlying reasons for poor adherence. For example, the World Health Organization's recent focus on digital technologies reflects our attention on individual-level determinants of adherence and reminder/observation-based systems[21]; far less research has addressed the social and structural barriers to staying on TB treatment.

In the UK, the development of an intervention to support adherence to treatment that is sensitive to the individual's cultural background and social circumstances, and can be routinely delivered within the NHS, is critical. To this purpose, a mixed-methods, patient-centred, approach to the study of the (modifiable factors that influence patients' adherence to treatment for TB is required.

Methods and analysis

Research question

Can a manualised package of intervention be developed to help overcome the social and cultural factors that lead to poor adherence to treatment in NHS patients in the United Kingdom with active TB?

Aim

To develop, pilot, and evaluate process and interim outcomes for an effective manualised intervention that improves the likelihood of adherence to treatment among NHS patients at risk of poor adherence due to social, cultural, and structural factors.

For peer review only - http://bmjopen.bmj.com/site/about/guidelines.xhtml

Objectives

1) Synthesise current knowledge on (a) determinants of adherence to treatment for TB, and (b) interventions that can support adherence, with particular emphasis on social and cultural barriers. (Scoping Review and Conceptual Framework)
2) Apply a conceptual framework of adherence endorsed by NICE guidelines (the Perceptions and Practicalities (PAPA) approach [18] to elucidate and address the personal, socio-cultural, and health systems context, mechanisms, and pathways of poor adherence among NHS patients with TB. (Formative Research)
3) Develop a manualised intervention (a series of systematic actions applied on the basis of a patient's needs assessment) with multiple components that can identify (a) NHS patients most at risk of non-adherence, (b) the salient modifiable barriers, and (c) the tailored support mechanisms required to meet individual patient needs by matching appropriate interventions to specific barriers, as recommended by NICE. (Development of Intervention) A manualised intervention was considered to be a suitable approach to managing adherence in TB, as it will enable a set of measures to be applied consistently within different NHS settings that will aid both clinicians and patients throughout the treatment journey. The content of the intervention will use existing support measures, employed in a systematic and structured way, and may also include any new interventions that are developed in response to the formative research.Pilot the intervention package in people at risk of poor adherence to define how the components work in combination and separately. (Pilot Study)
4) Evaluate the process of implementation of this intervention through describing the challenges and facilitators in delivering the package as intended (fidelity, reach) and assessing the impact of the intervention through evaluation of adherence indicators. (Process Evaluation)
5) Use the findings of the pilot to assess the costs of delivering the manualised intervention in an NHS setting, and to guide development of a proposal for a full randomised controlled trial (RCT).

Study design

This study uses a mixed methods approach. There are six sub-sections, reflecting the six objectives: i) Scoping Review and Conceptual Framework, ii) Formative Research, iii) Development of Intervention, iv) Pilot Study, v) Process Evaluation, vi) Cost Analysis and Future Work. Although the different elements are described separately below, the research activities for each will overlap, and some will run concurrently. The full programme of work and relationships between subsections is shown in Figure 1.

i. Scoping Review

To underpin the development of the manualised intervention, the first study sub-section will undertake literature reviews to answer the following research questions:

i) What personal, social, cultural, health systems-related and structural factors affect individuals' ability to adhere to treatment for TB?
ii) What kinds of intervention have been developed to address the multiple levels (personal, social, cultural, systems and structural) at which barriers to adherence may operate?
iii) What is the evidence for the successful impact of interventions to address barriers to adherence to treatment for TB?

These questions will be answered through the three following reviews:

For peer review only - http://bmjopen.bmj.com/site/about/guidelines.xhtml

1) A scoping review of qualitative studies that examine the personal, social, cultural, health systems-related and structural factors affecting adherence to treatment for TB from the perspectives of adult patients, care givers, or health care providers, as well as studies that evaluate interventions to support adherence to treatment for TB. Data from all settings will be considered, but with a particular focus on low incidence, high income, settings.

2) A critical review of quantitative studies examining the personal, social, cultural, health systems-related and structural factors affecting adherence to treatment for TB. This review focuses on low incidence, high income, settings and observational study designs. Given the scoping nature of the review, findings will be descriptively analysed and not be stratified or disaggregated e.g.by site of disease. All relative and absolute measures of effect will be extracted.

3) A critical review of quantitative studies that have examined the effectiveness of interventions to improve adherence in people taking treatment for TB, building on existing systematic reviews, including the provision and delivery of information and/or education; enablers and/or incentives; social support; case management approaches. Given the more limited literature, both observational studies and clinical trials will be included, from all settings. Findings will be descriptively analysed.

Together, these three reviews will enable us to conduct a critical interpretive synthesis of findings from qualitative and quantitative studies examining the assumptions and mechanisms of effect underlying interventions to improve adherence to treatment for TB.

ii. Formative Research

Formative research methods, recruitment and eligibility

In line with findings from the scoping review, we will develop topic guides for qualitative interviews exploring current models of TB service delivery in the study sites, providers' views on the barriers and facilitators for treatment adherence, and TB patients' and their family members' experiences of starting and staying on treatment. The thematic analysis of these interviews will complement insights gained from the scoping reviews which will allow us to refine a conceptual framework of adherence building on the Perceptions and Practicalities Approach (PAPA) [18]. Approximately 60 participants will be enrolled.

Adults (aged 18 and over) who are currently taking or recently completed treatment will be identified by the local TB service at four UK sites (Edinburgh, London [Central London & East London] and Southampton) and asked to take part in the study. The services have been chosen for their patient diversity, geographic spread and as reflecting the national TB picture. The patient group will be enriched with people who have been poorly adherent to treatment, although we will also include patients who report full adherence so that we can capture what may have enabled them to take treatment as prescribed. With patient consent, family members and/or carers will also be approached and asked to participate.

Health and social care workers from both primary and secondary care settings will be directly approached by researchers and invited to be interviewed. All those approached will be aged 18 or over and involved in TB management and care.

Data collection methods

Three different methods of data collection will be used to undertake the formative research.

For peer review only - http://bmjopen.bmj.com/site/about/guidelines.xhtml

1) **In-depth interviews – patients and family members/carers**

These will be conducted as individual interviews with approximately 30 participants, using a topic guide. The areas to be explored are: self-perception; personal beliefs and practices related to medicine-taking; health literacy and health-seeking behaviour; social support; cultural norms around health-seeking behaviour; financial and other structural barriers.

2) **Testing of BMQ and BIPQ Questionnaires with patients for their suitability**

To ensure that the validated questionnaires (i.e. Beliefs about Medicines Questionnaires[22], and the Brief Illness Perception Questionnaire[23], are accessible and acceptable to patients, we will also conduct cognitive interviews with 10 patients. We will then have confidence to use them in the later pilot study to assess patient perceptions and practicalities affecting adherence to anti-TB therapy. These will explore self-perception; personal beliefs; and practices related to medicine-taking.

3) **Semi-structured Interviews – healthcare providers**

These will be undertaken with health care providers responsible for multiple aspects of TB care (doctors, nurses, social workers, directly observed therapy [DOT] providers, managers and administrators). They will focus on providers' perceptions of factors affecting patient understanding of TB and its treatment; service delivery models including staffing; organisation of care; communication. We will interview four to six providers at each site, aiming for a total of 20 interviews.

We will use a framework approach [24] to facilitate initial analysis of the interview transcripts. Short patient case studies will be created for each patient interview. Data on health systems issues gained through mapping patient pathways and provider interviews will be organised using a deductive approach, with appropriate visual pathways.

iii. Development of the manualised intervention

Intervention development process

The data collected from the scoping reviews and formative research will be presented to, and synthesised by, an Intervention Development Group (IDG). A manualised intervention will be developed to identify (a) NHS patients most at risk of non-adherence, (b) their salient modifiable barriers, and (c) the tailored support mechanisms that meet individual patient needs by matching appropriate interventions to these specific barriers.

The IDG will include:

- patients (in particular the homeless, ethnic minorities, migrants new to the UK, and patients with drug resistant TB)
- family members/significant others of affected persons
- members of the public
- health care professionals (from both primary and secondary care)
- other professionals who work with patients/communities affected by TB

The constitution of the IDG will enable a co-production approach to ensure that the intervention is pragmatic, can be delivered within the context of existing care pathways, and is of benefit to service users and those who are likely to access the intervention.

Intervention contents

The manualised intervention is likely to consist of a screening and assessment tool, in addition to a package of measures that can be tailored to the needs of individual patients from different

For peer review only - http://bmjopen.bmj.com/site/about/guidelines.xhtml

population groups.

As patterns of adherence may be irregular over time, it is intended that the manualised intervention will be administered to all patients at each patient review. The tool thus needs to be quick and easy to administer. It may be electronically linked to patient records, enabling a comprehensive picture of the risk of possible non-adherence to be developed for each patient, as well as within a clinic population. The various delivery options for the intervention (such as using paper or an app) will be considered during its development stage.

The menu of supportive measures may, for example, include:

- Informational intervention: e.g. providing a convincing story setting out the rationale and on-going need for medication, addressing concerns about potential adverse effects and consequences of treatment and what to do if such events occur e.g. the participant will be informed that it is possible to change their treatment regimens to alternatives[25].
- Practicalities and Capability based interventions: video observed therapy [VOT], DOT, reminders including text messaging, automated methods for monitoring and feedback including electronic dosette boxes, use of a medication app, incentives e.g. financial and food vouchers, mitigation and management of drug toxicity due to treatments.
- Social and system interventions: offering flexibility in appointments; enhanced guidance on 'navigating' clinic pathways; signposting patients to relevant services, e.g. housing, drug and alcohol services, and social care; providing peer-support.

iv. Piloting the intervention

Study design, recruitment and eligibility

Once the intervention is developed, proof-of-concept is required within the real world. This will be undertaken using a cluster randomised pilot study that compares the manualised intervention to the usual standard of care in four London clinics treating TB. Two clinics will be randomly allocated to the intervention and two to standard of care. In the latter, the amount of support provided to patients is based on perceived need, as identified by a nurse-led review and a needs assessment. Most patients will have supported self-administered therapy, whilst others will be offered DOT and/or VOT if this is felt to be appropriate.

All consecutive patients aged 18 or over who are about to start treatment for TB, irrespective of site of disease, will be approached to take part in the study. We will exclude individuals who are unable to provide informed consent, those already on treatment and those who are not expected to live for the duration of the study (a minimum of six months from starting treatment). Within the pilot study, it is essential to capture the entire treatment period for each patient, in order to assess the effectiveness of the intervention. . Due to the nature of the TB patient population in the UK, patients are likely to include people at greater risk of poor adherence such as migrants newly arrived in the UK, people whose first language is not English, people with a mental health disorder, people taking immunosuppressive therapy or known to have immunodeficiency, those with a previous history of treatment for TB, or poor-adherence with anti-TB medication, and people with a current or previous history of drug or alcohol misuse.

The purpose of the pilot study is not formal hypothesis testing. Given this, a target sample size of 80 patients enrolled (20 per site) was identified for the pilot study as providing useful information that can help determine whether the intervention will be deliverable within a clinical setting. It will also guide the development of a possible larger definitive study using the intervention. The four TB clinics of interest (in East and North London) each treat in excess of 60 relevant patients per annum.

For peer review only - http://bmjopen.bmj.com/site/about/guidelines.xhtml

Based on usage of DOT within the clinic populations seen at the treatment sites (i.e. individuals currently identified as needing adherence support), we estimate that around 33% of patients will be at risk of non-adherence. Taking this as a minimum (as the manualised intervention is likely to be more sensitive than current risk assessments), we would expect that at least 26 of the 80 patients recruited will be identified as requiring adherence support. This sample size allows us to measure consent to enrolment for 80 individuals, data completeness for adherence and treatment outcomes for 80 individuals, data on acceptability and feasibility of the intervention package for around 40 individuals, and data on acceptability and feasibility of adherence support for at least 26 individuals (13 receiving the manualised intervention and 13 standard care).

Outcomes of the pilot The primary outcome of the study will be level of adherence, measured as the proportion of prescribed doses taken and assessed at six months from the start of treatment. In addition to this primary outcome, a number of secondary outcomes will also be measured, as follows:

1) Proportion consenting to the study.
2) Completeness of data for measures of adherence.
3) Proportion of patients withdrawing during the study and the reasons why.
4) Proportion of patients identified as needing adherence support in the intervention arm.
5) Proportion of patients offered adherence support and accepting it in the intervention arm.
6) Documentation of which adherence-promoting activities have been implemented among patients both in the standard of care and intervention arm, and when.
7) Detailed treatment implementation information: e.g. proportion of patients completing treatment, proportion of patients still on treatment after nine months or at study completion (whichever is the earlier).
8) Patterns of adherence (implementation and discontinuation).
9) Impact of manualised intervention on maintaining adherence over the duration of treatment.
10) Process variables – adherence-related perceptions and practicalities.

Measures of adherence
Our primary measure of adherence will be data obtained from medication monitoring boxes [26]. The boxes will not be set up act as a reminder system. Other measures will also be used and compared with this. These will include pill counts (the remaining medication in the box at the end of each month), and also patient-reported adherence, where we will ask patients to estimate how many doses they have missed in the last month. In the case of DOT or VOT methods being used, a record of missed doses will be kept.

Administration of the manualised intervention
The patient's case manager (usually the TB clinic nurse), plus a study research nurse, will apply the intervention in partnership with the patient to identify whether personal, socio-cultural and/or systems risk factors are present that suggest likely poor adherence with treatment. If these are identified, then the relevant measures outlined in the manualised intervention that may mitigate these will be reviewed and implemented with the agreement of the participant. These will be continued throughout the course of treatment, or stopped if no longer deemed to be relevant or required on reassessment.

Study schedule of visits
Study subjects will be seen at weeks 0, 2, 4, 8, 12, 16, 20 and 24 (earliest date of treatment completion). Should they require on-going treatment after six months, they will be seen as clinically indicated. At each review, adherence assessments will be performed in addition to an assessment of

For peer review only - http://bmjopen.bmj.com/site/about/guidelines.xhtml

perceptions and practicalities, the completion of the BMQ and the EQ-5D-5L Quality of Life questionnaires [22,27] and the GAD-7 [28] and PHQ-9[29] to assess anxiety and depression. The manualised intervention will be applied if the patient is attending a clinic that has been randomised to the intervention arm. All data will be collected by the research nurse, using standardised forms.

Follow Up

Most patients who do not have clinically important drug resistant disease receive six months of treatment. To allow for treatment interruptions, patients within the pilot study will be followed to either treatment completion or for a total of nine months from starting anti-TB therapy.

Analysis and interpretation

Where possible, univariable analyses will be undertaken to compare each outcome measure listed between study arms (intervention and control). For the primary outcome (adherence) the mean percentage value will be reported by arm (intervention and control) and histograms used to describe the distribution of values by study arm. Binary secondary outcomes will be reported by the proportion of individuals achieving this within each arm. The need to adjust for clustering by site and clinical care provider will be assessed using the cluster summary method (a t-test to compare the cluster means or proportions [as appropriate, two values per arm] between arms). An assessment of the balance in baseline characteristics between the study arms will also be conducted. If randomisation has failed to evenly distribute key characteristics (e.g. age, sex, ethnicity, or other factors identified as important during the scoping reviews), then the cluster means or proportions will be adjusted for these differences before applying the t-test. This two-stage approach to analysis is described by Hayes and Moulton [30]. We recognise that adherence data may be highly skewed and thus require compensatory analytical approaches.

The analysis of the first three of our secondary outcomes will address the feasibility of a definitive trial following a similar design to the pilot. Analysis of secondary outcomes four to six addresses the intervention, and complements the process evaluation (see below). Analysis of the primary outcome and final secondary outcomes around treatment adherence and completion provides initial information - given the modest sample size - concerning the effectiveness of the intervention, and may assist the sample size calculation for the definitive trial. They can also offer an alert in the unlikely event that the intervention is harmful.

Power calculation

Although we are undertaking a pilot study and thus the numbers enrolled are small, Table 1 indicates the power of our primary analysis to detect a range of absolute increases in adherence (10-30%) from a variety of baseline values (50-90%).

For peer review only - http://bmjopen.bmj.com/site/about/guidelines.xhtml

Table 1- Power calculation for the pilot study
Given a sample size of 80 individuals (40 per arm), across a range of baseline levels of adherence and absolute increases in that level, the pilot study has the following power.

Baseline adherence	Absolute increase	Power
70	30	0.98
60	30	0.88
50	30	0.82
80	20	0.86
70	20	0.61
60	20	0.49
90	10	0.54
80	10	0.24
70	10	0.18

v. Process evaluation

Evaluation method
We will evaluate the implementation process by analysing the challenges and facilitators in delivering the package. The impact of the intervention will be assessed by evaluation of adherence indicators. We will use the findings of the pilot to assess the costs of delivering the manualised intervention in an NHS setting, and to guide development of a proposal for a full RCT.

The process evaluation will consist of a description of the process of intervention implementation. It will assess how well the manualised intervention achieves its intended aim compared to standard care.

We will consider:
1) The fidelity of the intervention as delivered in comparison to how it was designed and envisaged,
2) The reach of the intervention (the proportion of the target group receiving it),
3) The barriers to facilitating implementation of the intervention and how these can be addressed,
4) The pre-existing factors that facilitated implementation.

Process measures, recruitment and eligibility
Process measures for each element of the package will be developed once the manual development has been completed, and used to assess success. They will include acceptability, uptake and change in practice. We will work with patients and staff separately at all four London sites. We will interview 20 patients (five at each study site i.e. 10 from each arm); and, if possible, 20 health care workers (five at each site). The patients will be selected within each site using purposeful sampling of clinic lists of every patient with active TB, to enable us to reflect the demographic spread of patients.

Key outputs will include a qualitative evaluation of delivery, the development of a narrative description of the process of intervention implementation and maintenance and a quantitative assessment of adherence-related perceptions and practicalities within intervention and control groups.

We will invite participation in the process evaluation from patients enrolled in the pilot study or staff

For peer review only - http://bmjopen.bmj.com/site/about/guidelines.xhtml

members treating patients at one of the four London sites also involved in delivering the pilot study. They will be included if they are aged 18 or over and able to provide informed consent. This will include probing anticipated versus real-life delivery of the intervention.

vi. Cost Analysis and Future Work

In order to generate realistic estimates of the cost of the intervention, cost data from the NHS perspective will be collected during the pilot study using a cost data collection tool used by health economists (The Client Service Receipt Inventory, CSRI), modified for TB [31].

After the pilot study and process evaluation have been completed, a final intervention package will be designed for use in a definitive RCT of the manualised package of interventions. The design of this final package will be based on the results of the process evaluation and the experience gained during the piloting of the intervention, modifying the definitive trial design and/or data collection accordingly.

Patient and public engagement

As documented above, patient representatives will sit on the IDG for the study. In addition, TB Alert, the UK's only national TB charity, has membership of the IDG. The role of the IDG, which will meet regularly throughout the study, is described in section iii. At the end of the study, the IDG will be involved in commenting on the findings and contributing to the dissemination plan.

Ethics and dissemination

This study received ethics approval on 24th December 2018 from Camberwell St Giles Ethics Committee (REC reference 18/LO/1818). Findings will be published and disseminated through peer-reviewed publications and conference presentations, published in an end of study report to our funder (the National Institute for Health Research, UK), and presented to key stakeholders.

Conclusion

Our study will develop and pilot a manualised intervention to improve adherence to treatment for TB in the UK using a mixed methods, patient-centred and provider-informed approach. This will enable us to begin to understand what motivates patients' treatment behaviour, whilst ensuring deliverability within the NHS. Our work is based on a robust understanding of the evidence on social, cultural and personal factors that influence adherence, and the interventions that are most effective in addressing these. The study reflects the geographic spread of TB in the UK and captures not only patient and expert clinical and academic experience, but also that of family and carers to develop the intervention. A key feature of the study is the co-production of a pragmatic and easy-to-use tool that utilises the best evidence on adherence, and allows its application in the clinic setting in a dynamic and iterative way.

Although the final pilot study may be limited to a relatively small sample size, it is hoped that its broad patient-centred perspective will make a useful contribution to our understanding of, and ability to deal effectively with, the risks of non-adherence to TB treatment in a population that can find this challenging. As many of the factors influencing adherence are likely to be generalisable to patients with other conditions in both high and low resource settings, this study also has the potential to inform adherence interventions in other disease areas.

For peer review only - http://bmjopen.bmj.com/site/about/guidelines.xhtml

Authors' contributions

ML, IA, RH, KK and HRS conceived of the work. HRS, IA, AC, RH, KK, MM, AS, NV, FW, HK, EP and ML designed the work. HRS, CNJC, MD, RH, KK, drafted the manuscript. All authors critically revised the manuscript and gave final approval of the version of the protocol manuscript to be published. All authors agree to be accountable for all aspects of the work in ensuring that questions related to the accuracy or integrity of any part are appropriately investigated and resolved.

Funding statement

This work was supported by the National Institute for Health Research (NIHR) Health Technology Assessment Programme, UK grant number 16/88/06.

The views expressed are those of the author(s) and not necessarily those of the National Health Service, UK, the NIHR or the Department of Health and Social Care.

This is an abridged version of the full study protocol, which can be accessed at https://www.fundingawards.nihr.ac.uk/award/16/88/06

Competing interests statement

IA, MD, HK, KK, ML, MM, EP, AS, FW have no competing interests to declare.

RH is supported by the National Institute for Health Research (NIHR, Collaboration for Leadership in Applied Health Research and Care (CLAHRC), North Thames at Bart's Health NHS Trust and Asthma UK (AUKCAR). Speaker engagements with honoraria with the following companies: Abbvie, Amgen, Astellas, AstraZeneca, Biogen, Erasmus, Idec, Gilead Sciences, GlaxoSmithKline, Janssen, Merck Sharp Dohme, Novartis, Pfizer, Roche, Shire Pharmaceuticals and TEVA. RH is Founding Director of a UCL-Business spin-out company (Spoonful of Sugar Ltd) providing consultancy on treatment engagement and patient support programmes to healthcare policy makers, providers and industry.

HRS reports grants from Medical Research Council (MRC), UK, grants from National Institute for Health Research (NIHR), UK, during the conduct of the study; other from Korean CDC and Johnson and Johnson (makers of Bedaquiline), other from Latvian Society Against Tuberculosis (funding through Otsuka and Johnson and Johnson), outside the submitted work.

CNJC reports personal fees from Public Health England, outside the submitted work.

For peer review only

For peer review only - http://bmjopen.bmj.com/site/about/guidelines.xhtml

References

1 Public Health England. Collaborative Tuberculosis Strategy for England 2015 to 2020. London: 2015. https://www.gov.uk/government/publications/collaborative-tuberculosis-strategy-for-england

2 Imperial MZ, Nahid P, Phillips PPJ, *et al.* A patient-level pooled analysis of treatment-shortening regimens for drug-susceptible pulmonary tuberculosis. *Nat Med* 2018;**24**:1708–15. doi:10.1038/s41591-018-0224-2

3 Kayigamba FR, Bakker MI, Mugisha V, *et al.* Adherence to Tuberculosis Treatment, Sputum Smear Conversion and Mortality: A Retrospective Cohort Study in 48 Rwandan Clinics. *PLoS One* 2013;**8**:e73501. doi:10.1371/journal.pone.0073501

4 Thomas A, Gopi PG, Santha T, *et al.* Predictors of relapse among pulmonary tuberculosis patients treated in a DOTS programme in South India. *Int J Tuberc Lung Dis* 2005;**9**:556–61.http://www.ncbi.nlm.nih.gov/pubmed/15875929 (accessed 1 May 2019).

5 Valencia S, León M, Losada I, *et al.* How do we measure adherence to anti-tuberculosis treatment? 2017;**15**:157–65. doi:10.1080/14787210.2017.1264270

6 van den Boogaard J, Boeree MJ, Kibiki GS, *et al.* The complexity of the adherence-response relationship in tuberculosis treatment: why are we still in the dark and how can we get out? *Trop Med Int Health* 2011;**16**:693–8. doi:10.1111/j.1365-3156.2011.02755.x

7 Zhdanov V, Bilenko N, Mor Z. Risk Factors for Recurrent Tuberculosis among Successfully Treated Patients in Israel, 1999-2011. *Isr Med Assoc J* 2017;**19**:237–41.http://www.ncbi.nlm.nih.gov/pubmed/28480678 (accessed 1 May 2019).

8 Bastard M, Sanchez-Padilla E, Hewison C, *et al.* Effects of treatment interruption patterns on treatment success among patients with multidrug-resistant tuberculosis in Armenia and Abkhazia. *J Infect Dis* 2015;**211**:1607–15. doi:10.1093/infdis/jiu551

9 Podewils LJ, Gler MTS, Quelapio MI, *et al.* Patterns of Treatment Interruption among Patients with Multidrug-Resistant TB (MDR TB) and Association with Interim and Final Treatment Outcomes. *PLoS One* 2013;**8**:e70064. doi:10.1371/journal.pone.0070064

10 Chirwa T, Nyasulu P, Chirwa E, *et al.* Levels of tuberculosis treatment adherence among sputum smear positive pulmonary tuberculosis patients attending care at Zomba Central hospital, southern Malawi, 2007-2008. *PLoS One* 2013;**8**:e63050. doi:10.1371/journal.pone.0063050

11 Arnold A, Cooke GS, Kon OM, *et al.* Drug resistant TB: UK multicentre study (DRUMS): Treatment, management and outcomes in London and West Midlands 2008–2014. *J Infect* 2017;**74**:260–71. doi:10.1016/j.jinf.2016.12.005

12 Pritchard AJ, Hayward AC, Monk PN, *et al.* Risk factors for drug resistant tuberculosis in Leicestershire--poor adherence to treatment remains an important cause of resistance. *Epidemiol Infect* 2003;**130**:481–3.http://www.ncbi.nlm.nih.gov/pubmed/12825732 (accessed 30 Jan 2019).

13 Cadosch D, Abel Zur Wiesch P, Kouyos R, *et al.* The Role of Adherence and Retreatment in De Novo Emergence of MDR-TB. *PLoS Comput Biol* 2016;**12**:e1004749. doi:10.1371/journal.pcbi.1004749

14 Saunders NJ, Trivedi UH, Thomson ML, *et al.* Deep resequencing of serial sputum isolates of Mycobacterium tuberculosis during therapeutic failure due to poor compliance reveals stepwise mutation of key resistance genes on an otherwise stable genetic background. *J Infect* 2011;**62**:212–7. doi:10.1016/j.jinf.2011.01.003

15 Shin SS, Keshavjee S, Gelmanova IY, *et al.* Development of Extensively Drug-resistant Tuberculosis during Multidrug-resistant Tuberculosis Treatment. *Am J Respir Crit Care Med* 2010;**182**:426–32. doi:10.1164/rccm.200911-1768OC

16 Bradford WZ, Martin JN, Reingold AL, *et al.* The changing epidemiology of acquired drug-resistant tuberculosis in San Francisco, USA. *Lancet* 1996;**348**:928–31. doi:10.1016/S0140-6736(96)03027-9

For peer review only - http://bmjopen.bmj.com/site/about/guidelines.xhtml

17 NICE. NICE Guideline: Tuberculosis. Natl. Inst. Heal. Care Excell. 2016;:178.
18 Horne R, Weinman J, Barber N, *et al.* Concordance, adherence and compliance in medicine taking. 2005. doi:10.1007/SpringerReference_64584
19 De Geest S, Sabaté E. Adherence to long-term therapies: Evidence for action. World Health Organization. Eur. J. Cardiovasc. Nurs. 2003. doi:10.1016/S1474-5151(03)00091-4
20 Vrijens B, De Geest S, Hughes DA, *et al.* A new taxonomy for describing and defining adherence to medications. *Br J Clin Pharmacol* 2012;**73**:691–705. doi:10.1111/j.1365-2125.2012.04167.x
21 World Health Organisation. *Handbook for the use of digital technologies to support tuberculosis medication adherence*. Geneva, Switzerland, Switzerland: 2017. https://www.who.int/tb/publications/2018/TB_medication_adherence_handbook_2018/en/
22 Horne R, Weinman J, Hankins M. The beliefs about medicines questionnaire: The development and evaluation of a new method for assessing the cognitive representation of medication. *Psychol Heal* 1999;**14**:1–24. doi:10.1080/08870449908407311
23 Broadbent E, Petrie KJ, Main J, *et al.* The Brief Illness Perception Questionnaire. *J Psychosom Res* 2006;**60**:631–7. doi:10.1016/j.jpsychores.2005.10.020
24 Gale NK, Heath G, Cameron E, *et al.* Using the framework method for the analysis of qualitative data in multi-disciplinary health research. *BMC Med Res Methodol* 2013;**13**:117. doi:10.1186/1471-2288-13-117
25 Michie S, van Stralen MM, West R. The behaviour change wheel: A new method for characterising and designing behaviour change interventions. *Implement Sci* 2011;**6**. doi:10.1186/1748-5908-6-42
26 Lewis JJ, Liu X, Zhang Z, *et al.* Evaluation of a medication monitor-based treatment strategy for drug-sensitive tuberculosis patients in China: study protocol for a cluster randomised controlled trial. *Trials* 2018;**19**:398. doi:10.1186/s13063-018-2650-3
27 Herdman M, Gudex C, Lloyd A, *et al.* Development and preliminary testing of the new five-level version of EQ-5D (EQ-5D-5L). *Qual Life Res* 2011;**20**:1727–36. doi:10.1007/s11136-011-9903-x
28 Spitzer RL, Kroenke K, Williams JBW, *et al.* A Brief Measure for Assessing Generalized Anxiety Disorder. *Arch Intern Med* 2006;**166**:1092. doi:10.1001/archinte.166.10.1092
29 Kroenke K, Spitzer RL, Williams JBW. The PHQ-9. *J Gen Intern Med* 2001;**16**:606–13. doi:10.1046/j.1525-1497.2001.016009606.x
30 Hayes RJ, Moulton LH, Moulton LH. *Cluster Randomised Trials, Second Edition*. Chapman and Hall/CRC 2017. doi:10.4324/9781315370286
31 Beecham J, Knapp M. 2 Costing psychiatric interventions. In: Graham, ed. *Measuring Mental Health Needs*. London: : Gaskell 2001. 200–24.http://www.rcpsych.ac.uk (accessed 12 Sep 2019).

For peer review only

For peer review only - http://bmjopen.bmj.com/site/about/guidelines.xhtml

Figure legends

Figure 1- The IMPACT study

The six mixed-methods elements of the IMPACT [Intervening with a Manualised Package to Achieve treatment adherence in people with Tuberculosis] study and the relationships between them. The study is guided by a steering group, which has oversight over the entire process and culminates in a pilot study with associated evaluatory elements.

For peer review only

For peer review only - http://bmjopen.bmj.com/site/about/guidelines.xhtml

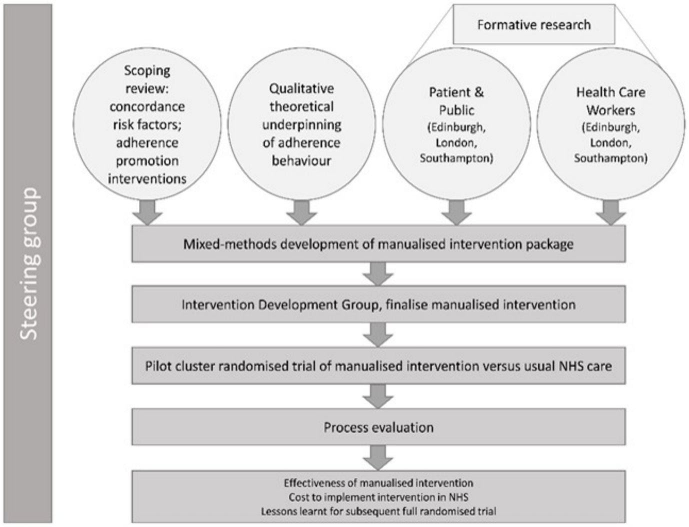

Figure 1- The IMPACT study
The six mixed-methods elements of the IMPACT [Intervening with a Manualised Package to Achieve treatment adherence in people with Tuberculosis] study and the relationships between them. The study is guided by a steering group, which has oversight over the entire process and culminates in a pilot study with associated evaluatory elements.

159x92mm (150 x 150 DPI)

BMJ Open

The IMPACT Study: Intervening with a Manualised Package to AChieve treatment adherence in people with Tuberculosis. A protocol paper for a mixed methods study, including a pilot randomised controlled trial

Journal:	*BMJ Open*
Manuscript ID	bmjopen-2019-032760.R2
Article Type:	Protocol
Date Submitted by the Author:	10-Oct-2019
Complete List of Authors:	Stagg, Helen R.; University of Edinburgh, Usher Institute<br>Abubakar, Ibrahim; University College London<br>Campbell, Colin; Public Health England, Tuberculosis Section, Centre for Infectious Disease Surveillance and Control, National Infection Service<br>Copas, Andrew; UCL<br>Darvell, Marcia; University College London<br>Horne, Robert; University College London<br>Kielmann, Karina; Queen Margaret University Edinburgh<br>Kunst, Heinke; Queen Mary University of London, Department of Respiratory Medicine<br>Mandelbaum, Mike; TB Alert<br>Pickett, Elisha; Royal Free London NHS Foundation Trust<br>Story, Alistair; University College Hospitals NHS Foundation Trust, Find&Treat<br>Vidal, Nicole; Queen Margaret University Edinburgh<br>Wurie, Fatima; University College London,<br>Lipman, Marc; Royal Free Campus, Respiratory Medicine
**Primary Subject Heading**:	Respiratory medicine
Secondary Subject Heading:	Patient-centred medicine, Infectious diseases, Epidemiology, Qualitative research
Keywords:	Tuberculosis < INFECTIOUS DISEASES, medication adherence, manuals, Protocols & guidelines < HEALTH SERVICES ADMINISTRATION & MANAGEMENT, pilot studies

SCHOLARONE™
Manuscripts

The IMPACT Study: Intervening with a Manualised Package to AChieve treatment adherence in people with Tuberculosis. A protocol paper for a mixed methods study, including a pilot randomised controlled trial

Author list: Stagg HR[1], Abubakar I[2], Campbell CNJ[2,3], Copas A[2], Darvell M[4], Horne R[5], Kielmann K[6], Kunst H[7], Mandelbaum M[8], Pickett E[9], Story A[10,11], Vidal N[6], Wurie F[3,12], Lipman M[4,9]

1. Usher Institute of Population Health Sciences and Informatics, The University of Edinburgh, MacKenzie House, 30 West Richmond Street, Edinburgh, EH8 9DX, UK
2. Institute for Global Health, University College London, 4th floor, Mortimer Market Centre, off Capper Street, London, WC1E 6JB, UK
3. TB Unit, National Infection Service, Public Health England, 61 Colindale Avenue, London NW9 5EQ, UK
4. UCL Respiratory, Division of Medicine, University College London, London, UK
5. Centre for Behavioural Medicine, UCL School of Pharmacy, Mezzanine Floor, BMA House, Tavistock Square, London, WC1H 9JP, UK
6. Institute for Global Health and Development, Queen Margaret University, Musselburgh, East Lothian, Edinburgh, EH21 6UU, UK
7. Blizard Institute, Barts and The London School of Medicine and Dentistry, Queen Mary University of London, London, E1 2AT, UK
8. TB Alert, Community Base, 113 Queens Road, Brighton, BN1 3XG, UK
9. Department of Respiratory Medicine, Royal Free London NHS Foundation Trust, Pond Street, London, NW3 2QG, UK
10. Collaborative Centre for Inclusion Health, University College London, London, UK
11. Find & Treat, University College London Hospitals NHS Foundation Trust, 235 Euston Road, London, NW1 2BU, UK
12. Department of Epidemiology and Public Health, Institute of Epidemiology and Health Care, University College London, UK

Corresponding author: Marc Lipman, Department of Respiratory Medicine, Royal Free London NHS Foundation Trust, Pond Street, London, NW3 2QG, UK; marclipman@nhs.net

Word Count: 4,544

Key words: tuberculosis, medication adherence, manuals, protocol, pilot studies

For peer review only - http://bmjopen.bmj.com/site/about/guidelines.xhtml

Abstract

Introduction

Compared to the rest of the UK and Western Europe, England has high rates of the infectious disease tuberculosis (TB). TB is curable, although treatment is for at least six months - and longer when disease is drug resistant. If patients miss too many doses (non-adherence), they may transmit infection for longer and the infecting bacteria may develop resistance to the standard drugs used for treatment. Non-adherence may therefore risk both their health and that of others. Within England, certain population groups are thought to be at higher risk of non-adherence, but the factors contributing to this have been insufficiently determined, as have the best interventions to promote adherence. The objective of this study is to develop a manualised package of interventions for use as part of routine care within National Health Services to address the social and cultural factors that lead to poor adherence to treatment for TB disease.

Methods and analysis

This study uses a mixed methods approach, with six study components. These are: (i) scoping reviews of the literature, (ii) qualitative research with patients, carers and healthcare professionals, (iii) development of the intervention, (iv) a pilot randomised controlled trial of the manualised intervention, (v) a process evaluation to examine clinical utility, and (vi) a cost analysis.

Ethics and dissemination

This study received ethics approval on 24th December 2018 from Camberwell St. Giles Ethics Committee, UK (REC reference 18/LO/1818). Findings will be published and disseminated through peer-reviewed publications and conference presentations, published in an end of study report to our funder (the National Institute for Health Research, UK), and presented to key stakeholders.

Trial Registration Number

ISRCTN registry, ISRCTN95243114, 14th February 2018

Secondary identifying numbers

University College London/University College London Hospitals Joint Research Office 17/0726
National Institute for Health Research, UK 16/88/06

For peer review only

For peer review only - http://bmjopen.bmj.com/site/about/guidelines.xhtml

Article Summary: Strengths and limitations of this study

Strengths

- Patient-centred, mixed methods approach, based on a robust understanding of the evidence on social, cultural and personal factors that influence adherence to medication to treat tuberculosis.
- Evidence and experience of adherence captured from a variety of perspectives from across the UK, including patients, their carers, and healthcare workers.
- Generalisable patient population of individuals at risk of non-adherence across low tuberculosis (TB) incidence settings.
- Development of a pragmatic and easy-to-use tool that captures the best evidence on adherence and allows its application in the clinic setting.

Limitations

- The study culminates in a pilot trial of the manualised intervention; a larger subsequent definitive trial is needed to test whether the intervention is efficacious and cost-effective beyond any initial conclusions regarding validity and feasibility derived from the pilot.

For peer review only

For peer review only - http://bmjopen.bmj.com/site/about/guidelines.xhtml

Introduction

Against a background of rising tuberculosis (TB) in the 1990s and 2000s, the need for a comprehensive approach to TB control in England was deemed necessary by Public Health England (PHE) and the National Health Service (NHS) England. In January 2015, these bodies jointly launched the 'Collaborative Tuberculosis Strategy for England 2015-2020'[1]. This seeks to reduce TB incidence, decrease health inequalities, and ultimately contribute to international efforts to eliminate TB as a public health problem. Ensuring that people can take all of their medication as prescribed is one of the strategy's priorities, as poor adherence to treatment for TB is a driver of worse patient outcomes[2–9], increases the risk of transmission (due to delayed sputum culture conversion)[10], and can promote the development of drug resistance[3,11–16]. Subsequent National Institute for Health and Care Excellence (NICE) guidance noted the lack of robust TB research in this area[17].

Barriers to optimal adherence to treatment for TB may occur for a number of reasons. These include:

- patient-related factors, including perceptions and beliefs,
- cultural influences and current mental state,
- structural economic factors and social support networks,
- health service factors that include treatment complexity as well as accessibility of those services and the relationships patients develop with service providers[18,19].

Non-adherence is not a single issue and may take various forms e.g. suboptimal implementation (skipping doses), or stopping treatment early (for example, as soon as a patient feels better; this is discontinuation)[20].

Although a series of studies have been undertaken to define the population groups most at risk of non-adherence[19], it is currently difficult, prior to starting medication, to identify who may struggle with taking treatment as prescribed. To date, methods to support treatment address some, but not all, of the important underlying reasons for poor adherence. For example, the World Health Organization's recent focus on digital technologies reflects our attention on individual-level determinants of adherence and reminder/observation-based systems[21]; far less research has addressed the social and structural barriers to staying on TB treatment.

In the UK, the development of an intervention to support adherence to treatment that is sensitive to the individual's cultural background and social circumstances, and can be routinely delivered within the NHS, is critical. To this purpose, a mixed-methods, patient-centred, approach to the study of the (modifiable factors that influence patients' adherence to treatment for TB is required.

Methods and analysis

Research question

Can a manualised package of intervention be developed to help overcome the social and cultural factors that lead to poor adherence to treatment in NHS patients in the United Kingdom with active TB?

Aim

To develop, pilot, and evaluate process and interim outcomes for an effective manualised intervention that improves the likelihood of adherence to treatment among NHS patients at risk of poor adherence due to social, cultural, and structural factors.

For peer review only - http://bmjopen.bmj.com/site/about/guidelines.xhtml

Objectives

1) Synthesise current knowledge on (a) determinants of adherence to treatment for TB, and (b) interventions that can support adherence, with particular emphasis on social and cultural barriers. (Scoping Review and Conceptual Framework)
2) Apply a conceptual framework of adherence endorsed by NICE guidelines (the Perceptions and Practicalities (PAPA) approach [18] to elucidate and address the personal, socio-cultural, and health systems context, mechanisms, and pathways of poor adherence among NHS patients with TB. (Formative Research)
3) Develop a manualised intervention (a series of systematic actions applied on the basis of a patient's needs assessment) with multiple components that can identify (a) NHS patients most at risk of non-adherence, (b) the salient modifiable barriers, and (c) the tailored support mechanisms required to meet individual patient needs by matching appropriate interventions to specific barriers, as recommended by NICE. (Development of Intervention) A manualised intervention was considered to be a suitable approach to managing adherence in TB, as it will enable a set of measures to be applied consistently within different NHS settings that will aid both clinicians and patients throughout the treatment journey. The content of the intervention will use existing support measures, employed in a systematic and structured way, and may also include any new interventions that are developed in response to the formative research. It will be compared to normal care.
4) Pilot the intervention package in people at risk of poor adherence to define how the components work in combination and separately. (Pilot Study)
5) Evaluate the process of implementation of this intervention through describing the challenges and facilitators in delivering the package as intended (fidelity, reach) and assessing the impact of the intervention through evaluation of adherence indicators. (Process Evaluation)
6) Use the findings of the pilot to assess the costs of delivering the manualised intervention in an NHS setting, and to guide development of a proposal for a full randomised controlled trial (RCT).

Study design

This study uses a mixed methods approach. There are six sub-sections, reflecting the six objectives: i) Scoping Review and Conceptual Framework, ii) Formative Research, iii) Development of Intervention, iv) Pilot Study, v) Process Evaluation, vi) Cost Analysis and Future Work. Although the different elements are described separately below, the research activities for each will overlap, and some will run concurrently. The full programme of work and relationships between subsections is shown in Figure 1. This paper reflects protocol version 4.0 (26th September 2019)[22].

i. Scoping Review

To underpin the development of the manualised intervention, the first study sub-section will undertake literature reviews to answer the following research questions:

i) What personal, social, cultural, health systems-related and structural factors affect individuals' ability to adhere to treatment for TB?
ii) What kinds of intervention have been developed to address the multiple levels (personal, social, cultural, systems and structural) at which barriers to adherence may operate?
iii) What is the evidence for the successful impact of interventions to address barriers to adherence to treatment for TB?

These questions will be answered through the three following reviews:

For peer review only - http://bmjopen.bmj.com/site/about/guidelines.xhtml

1) A scoping review of qualitative studies that examine the personal, social, cultural, health systems-related and structural factors affecting adherence to treatment for TB from the perspectives of adult patients, care givers, or health care providers, as well as studies that evaluate interventions to support adherence to treatment for TB. Data from all settings will be considered, but with a particular focus on low incidence, high income, settings.

2) A critical review of quantitative studies examining the personal, social, cultural, health systems-related and structural factors affecting adherence to treatment for TB. This review focuses on low incidence, high income, settings and observational study designs. Given the scoping nature of the review, findings will be descriptively analysed and not be stratified or disaggregated e.g.by site of disease. All relative and absolute measures of effect will be extracted.

3) A critical review of quantitative studies that have examined the effectiveness of interventions to improve adherence in people taking treatment for TB, building on existing systematic reviews, including the provision and delivery of information and/or education; enablers and/or incentives; social support; case management approaches. Given the more limited literature, both observational studies and clinical trials will be included, from all settings. Findings will be descriptively analysed.

Together, these three reviews will enable us to conduct a critical interpretive synthesis of findings from qualitative and quantitative studies examining the assumptions and mechanisms of effect underlying interventions to improve adherence to treatment for TB. Further details on the reviews are available in the full protocol[22].

ii. Formative Research

Formative research methods, recruitment and eligibility

In line with findings from the scoping review, we will develop topic guides for qualitative interviews exploring current models of TB service delivery in the study sites, providers' views on the barriers and facilitators for treatment adherence, and TB patients' and their family members' experiences of starting and staying on treatment. Details about these guides are available within the full protocol[22]. The thematic analysis of these interviews will complement insights gained from the scoping reviews which will allow us to refine a conceptual framework of adherence building on the Perceptions and Practicalities Approach (PAPA) [18]. Approximately 60 participants will be enrolled.

Adults (aged 18 and over) who are currently taking or recently completed treatment will be identified by the local TB service at four UK sites (Edinburgh, London [Central London & East London] and Southampton) and asked to take part in the study. The services have been chosen for their patient diversity, geographic spread and as reflecting the national TB picture. The patient group will be enriched with people who have been poorly adherent to treatment, although we will also include patients who report full adherence so that we can capture what may have enabled them to take treatment as prescribed. With patient consent, family members and/or carers will also be approached and asked to participate.

Health and social care workers from both primary and secondary care settings will be directly approached by researchers and invited to be interviewed. All those approached will be aged 18 or over and involved in TB management and care.

Data collection methods

Three different methods of data collection will be used to undertake the formative research.

For peer review only - http://bmjopen.bmj.com/site/about/guidelines.xhtml

1) In-depth interviews – patients and family members/carers

These will be conducted as individual interviews with approximately 30 participants, using a topic guide. The areas to be explored are: self-perception; personal beliefs and practices related to medicine-taking; health literacy and health-seeking behaviour; social support; cultural norms around health-seeking behaviour; financial and other structural barriers.

2) Testing of BMQ and BIPQ Questionnaires with patients for their suitability

To ensure that the validated questionnaires (i.e. Beliefs about Medicines Questionnaires[23], and the Brief Illness Perception Questionnaire[24], are accessible and acceptable to patients, we will also conduct cognitive interviews with 10 patients. We will then have confidence to use them in the later pilot study to assess patient perceptions and practicalities affecting adherence to anti-TB therapy. These will explore self-perception; personal beliefs; and practices related to medicine-taking.

3) Semi-structured interviews – healthcare providers

These will be undertaken with health care providers responsible for multiple aspects of TB care (doctors, nurses, social workers, directly observed therapy [DOT] providers, managers and administrators). They will focus on providers' perceptions of factors affecting patient understanding of TB and its treatment; service delivery models including staffing; organisation of care; communication. We will interview four to six providers at each site, aiming for a total of 20 interviews.

We will use a framework approach [25] to facilitate initial analysis of the interview transcripts. Short patient case studies will be created for each patient interview. Data on health systems issues gained through mapping patient pathways and provider interviews will be organised using a deductive approach, with appropriate visual pathways.

iii. Development of the manualised intervention

Intervention development process

The data collected from the scoping reviews and formative research will be presented to, and synthesised by, an Intervention Development Group (IDG). A manualised intervention will be developed to identify (a) NHS patients most at risk of non-adherence, (b) their salient modifiable barriers, and (c) the tailored support mechanisms that meet individual patient needs by matching appropriate interventions to these specific barriers.

The IDG will include:

- patients (in particular the homeless, ethnic minorities, migrants new to the UK, and patients with drug resistant TB)
- family members/significant others of affected persons
- members of the public
- health care professionals (from both primary and secondary care)
- other professionals who work with patients/communities affected by TB

The constitution of the IDG will enable a co-production approach to ensure that the intervention is pragmatic, can be delivered within the context of existing care pathways, and is of benefit to service users and those who are likely to access the intervention.

Intervention contents

The manualised intervention is likely to consist of a screening and assessment tool, in addition to a package of measures that can be tailored to the needs of individual patients from different population groups.

For peer review only - http://bmjopen.bmj.com/site/about/guidelines.xhtml

As patterns of adherence may be irregular over time, it is intended that the manualised intervention will be administered to all patients at each patient review. The tool thus needs to be quick and easy to administer. It may be electronically linked to patient records, enabling a comprehensive picture of the risk of possible non-adherence to be developed for each patient, as well as within a clinic population. The various delivery options for the intervention (such as using paper or an app) will be considered during its development stage.

The menu of supportive measures may, for example, include:

- Informational intervention: e.g. providing a convincing story setting out the rationale and on-going need for medication, addressing concerns about potential adverse effects and consequences of treatment and what to do if such events occur e.g. the participant will be informed that it is possible to change their treatment regimens to alternatives[26].
- Practicalities and Capability based interventions: video observed therapy [VOT], DOT, reminders including text messaging, automated methods for monitoring and feedback including electronic dosette boxes, use of a medication app, incentives e.g. financial and food vouchers, mitigation and management of drug toxicity due to treatments.
- Social and system interventions: offering flexibility in appointments; enhanced guidance on 'navigating' clinic pathways; signposting patients to relevant services, e.g. housing, drug and alcohol services, and social care; providing peer-support.

iv. Piloting the intervention

Study design, recruitment and eligibility

Once the intervention is developed, proof-of-concept is required within the real world. This will be undertaken using a non-blinded cluster randomised pilot study that compares the manualised intervention to the usual standard of care in four London clinics treating TB. Two clinics will be randomly allocated to the intervention and two to standard of care. In the latter, the amount of support provided to patients is based on perceived need, as identified by a nurse-led review and a needs assessment. Most patients will have supported self-administered therapy, whilst others will be offered DOT and/or VOT if this is felt to be appropriate. We anticipate enrolment to commence in January 2020 (recruitment is pending).

All consecutive patients aged 18 or over who are about to start treatment for TB, irrespective of site of disease, will be approached to take part in the study. We will exclude individuals who are unable to provide informed consent, those already on treatment and those who are not expected to live for the duration of the study (a minimum of six months from starting treatment). Within the pilot study, it is essential to capture the entire treatment period for each patient, in order to assess the effectiveness of the intervention. Due to the nature of the TB patient population in the UK, patients are likely to include people at greater risk of poor adherence such as migrants newly arrived in the UK, people whose first language is not English, people with a mental health disorder, people taking immunosuppressive therapy or known to have immunodeficiency, those with a previous history of treatment for TB, or poor-adherence with anti-TB medication, and people with a current or previous history of drug or alcohol misuse.

The purpose of the pilot study is not formal hypothesis testing. Given this, a target sample size of 80 patients enrolled (20 per site) was identified for the pilot study as providing useful information that can help determine whether the intervention will be deliverable within a clinical setting. It will also guide the development of a possible larger definitive study using the intervention. The four TB clinics of interest (in East and North London) each treat in excess of 60 relevant patients per annum.

Based on usage of DOT within the clinic populations seen at the treatment sites (i.e. individuals

For peer review only - http://bmjopen.bmj.com/site/about/guidelines.xhtml

currently identified as needing adherence support), we estimate that around 33% of patients will be at risk of non-adherence. Taking this as a minimum (as the manualised intervention is likely to be more sensitive than current risk assessments), we would expect that at least 26 of the 80 patients recruited will be identified as requiring adherence support. This sample size allows us to measure consent to enrolment for 80 individuals, data completeness for adherence and treatment outcomes for 80 individuals, data on acceptability and feasibility of the intervention package for around 40 individuals, and data on acceptability and feasibility of adherence support for at least 26 individuals (13 receiving the manualised intervention and 13 standard care).

Outcomes of the pilot

The primary outcome of the study will be level of adherence, measured as the proportion of prescribed doses taken and assessed at six months from the start of treatment. In addition to this primary outcome, a number of secondary outcomes will also be measured, as follows:

1) Proportion consenting to the study.
2) Completeness of data for measures of adherence.
3) Proportion of patients withdrawing during the study and the reasons why.
4) Proportion of patients identified as needing adherence support in the intervention arm.
5) Proportion of patients offered adherence support and accepting it in the intervention arm.
6) Documentation of which adherence-promoting activities have been implemented among patients both in the standard of care and intervention arm, and when.
7) Detailed treatment implementation information: e.g. proportion of patients completing treatment, proportion of patients still on treatment after nine months or at study completion (whichever is the earlier).
8) Patterns of adherence (implementation and discontinuation).
9) Impact of manualised intervention on maintaining adherence over the duration of treatment.
10) Process variables – adherence-related perceptions and practicalities.

Measures of adherence

Our primary measure of adherence will be data obtained from medication monitoring boxes [27]. The boxes will not be set up act as a reminder system. Other measures will also be used and compared with this. These will include pill counts (the remaining medication in the box at the end of each month), and also patient-reported adherence, where we will ask patients to estimate how many doses they have missed in the last month. In the case of DOT or VOT methods being used, a record of missed doses will be kept.

Administration of the manualised intervention

The patient's case manager (usually the TB clinic nurse), plus a study research nurse, will apply the intervention in partnership with the patient to identify whether personal, socio-cultural and/or systems risk factors are present that suggest likely poor adherence with treatment. If these are identified, then the relevant measures outlined in the manualised intervention that may mitigate these will be reviewed and implemented with the agreement of the participant. These will be continued throughout the course of treatment, or stopped if no longer deemed to be relevant or required on reassessment.

Study schedule of visits

Study subjects will be seen at weeks 0, 2, 4, 8, 12, 16, 20 and 24 (earliest date of treatment completion). Should they require on-going treatment after six months, they will be seen as clinically indicated. At each review, adherence assessments will be performed in addition to an assessment of perceptions and practicalities, the completion of the BMQ and the EQ-5D-5L Quality of Life

For peer review only - http://bmjopen.bmj.com/site/about/guidelines.xhtml

questionnaires [23,28] and the GAD-7 [29] and PHQ-9[30] to assess anxiety and depression. The manualised intervention will be applied if the patient is attending a clinic that has been randomised to the intervention arm. All data will be collected by the research nurse, using standardised forms.

Follow Up

Most patients who do not have clinically important drug resistant disease receive six months of treatment. To allow for treatment interruptions, patients within the pilot study will be followed to either treatment completion or for a total of nine months from starting anti-TB therapy.

Analysis and interpretation

Where possible, univariable analyses will be undertaken to compare each outcome measure listed between study arms (intervention and control). For the primary outcome (adherence) the mean percentage value will be reported by arm (intervention and control) and histograms used to describe the distribution of values by study arm. Binary secondary outcomes will be reported by the proportion of individuals achieving the outcome within each arm. The need to adjust for clustering by site and clinical care provider will be assessed using the cluster summary method (a t-test to compare the cluster means or proportions [as appropriate, two values per arm] between arms). An assessment of the balance in baseline characteristics between the study arms will also be conducted. If randomisation has failed to evenly distribute key characteristics (e.g. age, sex, ethnicity, or other factors identified as important during the scoping reviews), then the cluster means or proportions will be adjusted for these differences before applying the t-test. This two-stage approach to analysis is described by Hayes and Moulton [31]. We recognise that adherence data may be highly skewed and thus require compensatory analytical approaches.

The analysis of the first three of our secondary outcomes will address the feasibility of a definitive trial following a similar design to the pilot. Analysis of secondary outcomes four to six addresses the intervention, and complements the process evaluation (see below). Analysis of the primary outcome and final secondary outcomes around treatment adherence and completion provides initial information - given the modest sample size - concerning the effectiveness of the intervention, and may assist the sample size calculation for the definitive trial. They can also offer an alert in the unlikely event that the intervention is harmful.

Power calculation

Although we are undertaking a pilot study and thus the numbers enrolled are small, Table 1 indicates the power of our primary analysis to detect a range of absolute increases in adherence (10-30%) from a variety of baseline values (50-90%).

For peer review only - http://bmjopen.bmj.com/site/about/guidelines.xhtml

Table 1- Power calculation for the pilot study
Given a sample size of 80 individuals (40 per arm), across a range of baseline levels of adherence and absolute increases in that level, the pilot study has the following power.

Baseline adherence	Absolute increase	Power
70	30	0.98
60	30	0.88
50	30	0.82
80	20	0.86
70	20	0.61
60	20	0.49
90	10	0.54
80	10	0.24
70	10	0.18

v. Process evaluation

Evaluation method
We will evaluate the implementation process by analysing the challenges and facilitators in delivering the package. The impact of the intervention will be assessed by evaluation of adherence indicators. We will use the findings of the pilot to assess the costs of delivering the manualised intervention in an NHS setting, and to guide development of a proposal for a full RCT.

The process evaluation will consist of a description of the process of intervention implementation. It will assess how well the manualised intervention achieves its intended aim compared to standard care.

We will consider:
1) The fidelity of the intervention as delivered in comparison to how it was designed and envisaged,
2) The reach of the intervention (the proportion of the target group receiving it),
3) The barriers to facilitating implementation of the intervention and how these can be addressed,
4) The pre-existing factors that facilitated implementation.

Process measures, recruitment and eligibility
Process measures for each element of the package will be developed once the manual development has been completed, and used to assess success. They will include acceptability, uptake and change in practice. We will work with patients and staff separately at all four London sites. We will interview 20 patients (five at each study site i.e. 10 from each arm); and, if possible, 20 health care workers (five at each site). The patients will be selected within each site using purposeful sampling of clinic lists of every patient with active TB, to enable us to reflect the demographic spread of patients.

Key outputs will include a qualitative evaluation of delivery, the development of a narrative description of the process of intervention implementation and maintenance and a quantitative assessment of adherence-related perceptions and practicalities within intervention and control groups.

We will invite participation in the process evaluation from patients enrolled in the pilot study or staff

For peer review only - http://bmjopen.bmj.com/site/about/guidelines.xhtml

members treating patients at one of the four London sites also involved in delivering the pilot study. They will be included if they are aged 18 or over and able to provide informed consent. This will include probing anticipated versus real-life delivery of the intervention.

vi. Cost Analysis and Future Work

In order to generate realistic estimates of the cost of the intervention, cost data from the NHS perspective will be collected during the pilot study using a cost data collection tool used by health economists (The Client Service Receipt Inventory, CSRI), modified for TB [32].

After the pilot study and process evaluation have been completed, a final intervention package will be designed for use in a definitive RCT of the manualised package of interventions. The design of this final package will be based on the results of the process evaluation and the experience gained during the piloting of the intervention, modifying the definitive trial design and/or data collection accordingly.

Patient and public engagement

As documented above, patient representatives will sit on the IDG for the study. In addition, TB Alert, the UK's only national TB charity, has membership of the IDG. The role of the IDG, which will meet regularly throughout the study, is described in section iii. At the end of the study, the IDG will be involved in commenting on the findings and contributing to the dissemination plan.

Ethics, sponsorship, contact details and dissemination

The study is sponsored by the Joint Research Office of University College London and University College London Hospitals. This study received ethics approval on 24th December 2018 from Camberwell St Giles Ethics Committee (REC reference 18/LO/1818; Level 3, Block B, Whitefriars, Lewins Mead, Bristol, BS1 2NT, UK; +44 (0)207104 8204; NRESCommittee.London-CamberwellStGiles@nhs.net). Findings will be published and disseminated through peer-reviewed publications and conference presentations, published in an end of study report to our funder (the National Institute for Health Research, UK), and presented to key stakeholders.

For public enquiries, please contact Marcia Darvell, IMPACT Study Project Co-ordinator, Respiratory Medicine, The Royal Free London NHS Foundation Trust, UCL Medical School Building, Rowland Hill Street, London, NW3 2PF, UK; +4420 8016 8375; m.darvell@ucl.ac.uk

For scientific enquiries, please contact the Chief Investigator Professor Marc Lipman (The Royal Free London NHS Foundation Trust and University College London), Respiratory Medicine, The Grove Centre, The Royal Free London NHS Foundation Trust, Rowland Hill Street, London, NW3 2PF, UK; +4420 7472 6452; marclipman@nhs.net.

Data sharing statement

The datasets generated during and/or analysed during the current study will be available upon request in a de-identified format and after publication of study outcomes and associated permission from the funder. Requests for data should be directed to Professor Marc Lipman as per the contact details above.

Conclusion

Our study will develop and pilot a manualised intervention to improve adherence to treatment for

For peer review only - http://bmjopen.bmj.com/site/about/guidelines.xhtml

TB in the UK using a mixed methods, patient-centred and provider-informed approach. This will enable us to begin to understand what motivates patients' treatment behaviour, whilst ensuring deliverability within the NHS. Our work is based on a robust understanding of the evidence on social, cultural and personal factors that influence adherence, and the interventions that are most effective in addressing these. The study reflects the geographic spread of TB in the UK and captures not only patient and expert clinical and academic experience, but also that of family and carers to develop the intervention. A key feature of the study is the co-production of a pragmatic and easy-to-use tool that utilises the best evidence on adherence, and allows its application in the clinic setting in a dynamic and iterative way.

Although the final pilot study may be limited to a relatively small sample size, it is hoped that its broad patient-centred perspective will make a useful contribution to our understanding of, and ability to deal effectively with, the risks of non-adherence to TB treatment in a population that can find this challenging. As many of the factors influencing adherence are likely to be generalisable to patients with other conditions in both high and low resource settings, this study also has the potential to inform adherence interventions in other disease areas.

For peer review only

For peer review only - http://bmjopen.bmj.com/site/about/guidelines.xhtml

Authors' contributions

ML, IA, RH, KK and HRS conceived of the work. HRS, IA, AC, RH, KK, MM, AS, NV, FW, HK, EP and ML designed the work. HRS, CNJC, MD, RH, KK, drafted the manuscript. All authors critically revised the manuscript and gave final approval of the version of the protocol manuscript to be published. All authors agree to be accountable for all aspects of the work in ensuring that questions related to the accuracy or integrity of any part are appropriately investigated and resolved.

Funding statement

This work was supported by the National Institute for Health Research (NIHR) Health Technology Assessment Programme, UK grant number 16/88/06.

The views expressed are those of the author(s) and not necessarily those of the National Health Service, UK, the NIHR or the Department of Health and Social Care.

This is an abridged version of the full study protocol, which can be accessed at https://www.fundingawards.nihr.ac.uk/award/16/88/06

Competing interests statement

IA, MD, HK, KK, ML, MM, EP, AS, FW have no competing interests to declare.

RH is supported by the National Institute for Health Research (NIHR, Collaboration for Leadership in Applied Health Research and Care (CLAHRC), North Thames at Bart's Health NHS Trust and Asthma UK (AUKCAR). Speaker engagements with honoraria with the following companies: Abbvie, Amgen, Astellas, AstraZeneca, Biogen, Erasmus, Idec, Gilead Sciences, GlaxoSmithKline, Janssen, Merck Sharp Dohme, Novartis, Pfizer, Roche, Shire Pharmaceuticals and TEVA. RH is Founding Director of a UCL-Business spin-out company (Spoonful of Sugar Ltd) providing consultancy on treatment engagement and patient support programmes to healthcare policy makers, providers and industry.

HRS reports grants from Medical Research Council (MRC), UK, grants from National Institute for Health Research (NIHR), UK, during the conduct of the study; other from Korean CDC and Johnson and Johnson (makers of Bedaquiline), other from Latvian Society Against Tuberculosis (funding through Otsuka and Johnson and Johnson), outside the submitted work.

CNJC reports personal fees from Public Health England, outside the submitted work.

For peer review only

For peer review only - http://bmjopen.bmj.com/site/about/guidelines.xhtml

References

1 Public Health England. Collaborative Tuberculosis Strategy for England 2015 to 2020. London: 2015. https://www.gov.uk/government/publications/collaborative-tuberculosis-strategy-for-england

2 Imperial MZ, Nahid P, Phillips PPJ, *et al.* A patient-level pooled analysis of treatment-shortening regimens for drug-susceptible pulmonary tuberculosis. *Nat Med* 2018;**24**:1708–15. doi:10.1038/s41591-018-0224-2

3 Kayigamba FR, Bakker MI, Mugisha V, *et al.* Adherence to Tuberculosis Treatment, Sputum Smear Conversion and Mortality: A Retrospective Cohort Study in 48 Rwandan Clinics. *PLoS One* 2013;**8**:e73501. doi:10.1371/journal.pone.0073501

4 Thomas A, Gopi PG, Santha T, *et al.* Predictors of relapse among pulmonary tuberculosis patients treated in a DOTS programme in South India. *Int J Tuberc Lung Dis* 2005;**9**:556–61.http://www.ncbi.nlm.nih.gov/pubmed/15875929 (accessed 1 May 2019).

5 Valencia S, León M, Losada I, *et al.* How do we measure adherence to anti-tuberculosis treatment? 2017;**15**:157–65. doi:10.1080/14787210.2017.1264270

6 van den Boogaard J, Boeree MJ, Kibiki GS, *et al.* The complexity of the adherence-response relationship in tuberculosis treatment: why are we still in the dark and how can we get out? *Trop Med Int Health* 2011;**16**:693–8. doi:10.1111/j.1365-3156.2011.02755.x

7 Zhdanov V, Bilenko N, Mor Z. Risk Factors for Recurrent Tuberculosis among Successfully Treated Patients in Israel, 1999-2011. *Isr Med Assoc J* 2017;**19**:237–41.http://www.ncbi.nlm.nih.gov/pubmed/28480678 (accessed 1 May 2019).

8 Bastard M, Sanchez-Padilla E, Hewison C, *et al.* Effects of treatment interruption patterns on treatment success among patients with multidrug-resistant tuberculosis in Armenia and Abkhazia. *J Infect Dis* 2015;**211**:1607–15. doi:10.1093/infdis/jiu551

9 Podewils LJ, Gler MTS, Quelapio MI, *et al.* Patterns of Treatment Interruption among Patients with Multidrug-Resistant TB (MDR TB) and Association with Interim and Final Treatment Outcomes. *PLoS One* 2013;**8**:e70064. doi:10.1371/journal.pone.0070064

10 Chirwa T, Nyasulu P, Chirwa E, *et al.* Levels of tuberculosis treatment adherence among sputum smear positive pulmonary tuberculosis patients attending care at Zomba Central hospital, southern Malawi, 2007-2008. *PLoS One* 2013;**8**:e63050. doi:10.1371/journal.pone.0063050

11 Arnold A, Cooke GS, Kon OM, *et al.* Drug resistant TB: UK multicentre study (DRUMS): Treatment, management and outcomes in London and West Midlands 2008–2014. *J Infect* 2017;**74**:260–71. doi:10.1016/j.jinf.2016.12.005

12 Pritchard AJ, Hayward AC, Monk PN, *et al.* Risk factors for drug resistant tuberculosis in Leicestershire--poor adherence to treatment remains an important cause of resistance. *Epidemiol Infect* 2003;**130**:481–3.http://www.ncbi.nlm.nih.gov/pubmed/12825732 (accessed 30 Jan 2019).

13 Cadosch D, Abel Zur Wiesch P, Kouyos R, *et al.* The Role of Adherence and Retreatment in De Novo Emergence of MDR-TB. *PLoS Comput Biol* 2016;**12**:e1004749. doi:10.1371/journal.pcbi.1004749

14 Saunders NJ, Trivedi UH, Thomson ML, *et al.* Deep resequencing of serial sputum isolates of Mycobacterium tuberculosis during therapeutic failure due to poor compliance reveals stepwise mutation of key resistance genes on an otherwise stable genetic background. *J Infect* 2011;**62**:212–7. doi:10.1016/j.jinf.2011.01.003

15 Shin SS, Keshavjee S, Gelmanova IY, *et al.* Development of Extensively Drug-resistant Tuberculosis during Multidrug-resistant Tuberculosis Treatment. *Am J Respir Crit Care Med* 2010;**182**:426–32. doi:10.1164/rccm.200911-1768OC

16 Bradford WZ, Martin JN, Reingold AL, *et al.* The changing epidemiology of acquired drug-resistant tuberculosis in San Francisco, USA. *Lancet* 1996;**348**:928–31. doi:10.1016/S0140-6736(96)03027-9

For peer review only - http://bmjopen.bmj.com/site/about/guidelines.xhtml

17 NICE. NICE Guideline: Tuberculosis. Natl. Inst. Heal. Care Excell. 2016;:178.

18 Horne R, Weinman J, Barber N, *et al.* Concordance, adherence and compliance in medicine taking. 2005. doi:10.1007/SpringerReference_64584

19 De Geest S, Sabaté E. Adherence to long-term therapies: Evidence for action. World Health Organization. Eur. J. Cardiovasc. Nurs. 2003. doi:10.1016/S1474-5151(03)00091-4

20 Vrijens B, De Geest S, Hughes DA, *et al.* A new taxonomy for describing and defining adherence to medications. *Br J Clin Pharmacol* 2012;**73**:691–705. doi:10.1111/j.1365-2125.2012.04167.x

21 World Health Organisation. *Handbook for the use of digital technologies to support tuberculosis medication adherence*. Geneva, Switzerland, Switzerland: 2017. https://www.who.int/tb/publications/2018/TB_medication_adherence_handbook_2018/en/

22 National Institute for Health Research (NIHR). NIHR Research Award: Intervening with a Manualised Package to achieve treatment adherence in people with Tuberculosis: the IMPACT study. https://fundingawards.nihr.ac.uk/award/16/88/06 (accessed 9 Oct 2019).

23 Horne R, Weinman J, Hankins M. The beliefs about medicines questionnaire: The development and evaluation of a new method for assessing the cognitive representation of medication. *Psychol Heal* 1999;**14**:1–24. doi:10.1080/08870449908407311

24 Broadbent E, Petrie KJ, Main J, *et al.* The Brief Illness Perception Questionnaire. *J Psychosom Res* 2006;**60**:631–7. doi:10.1016/j.jpsychores.2005.10.020

25 Gale NK, Heath G, Cameron E, *et al.* Using the framework method for the analysis of qualitative data in multi-disciplinary health research. *BMC Med Res Methodol* 2013;**13**:117. doi:10.1186/1471-2288-13-117

26 Michie S, van Stralen MM, West R. The behaviour change wheel: A new method for characterising and designing behaviour change interventions. *Implement Sci* 2011;**6**. doi:10.1186/1748-5908-6-42

27 Lewis JJ, Liu X, Zhang Z, *et al.* Evaluation of a medication monitor-based treatment strategy for drug-sensitive tuberculosis patients in China: study protocol for a cluster randomised controlled trial. *Trials* 2018;**19**:398. doi:10.1186/s13063-018-2650-3

28 Herdman M, Gudex C, Lloyd A, *et al.* Development and preliminary testing of the new five-level version of EQ-5D (EQ-5D-5L). *Qual Life Res* 2011;**20**:1727–36. doi:10.1007/s11136-011-9903-x

29 Spitzer RL, Kroenke K, Williams JBW, *et al.* A Brief Measure for Assessing Generalized Anxiety Disorder. *Arch Intern Med* 2006;**166**:1092. doi:10.1001/archinte.166.10.1092

30 Kroenke K, Spitzer RL, Williams JBW. The PHQ-9. *J Gen Intern Med* 2001;**16**:606–13. doi:10.1046/j.1525-1497.2001.016009606.x

31 Hayes RJ, Moulton LH, Moulton LH. *Cluster Randomised Trials, Second Edition*. Chapman and Hall/CRC 2017. doi:10.4324/9781315370286

32 Beecham J, Knapp M. 2 Costing psychiatric interventions. In: Graham, ed. *Measuring Mental Health Needs*. London: : Gaskell 2001. 200–24.http://www.rcpsych.ac.uk (accessed 12 Sep 2019).

For peer review only - http://bmjopen.bmj.com/site/about/guidelines.xhtml

Figure legends

Figure 1- The IMPACT study
The six mixed-methods elements of the IMPACT [Intervening with a Manualised Package to Achieve treatment adherence in people with Tuberculosis] study and the relationships between them. The study is guided by a steering group, which has oversight over the entire process and culminates in a pilot study with associated evaluatory elements.

For peer review only

For peer review only - http://bmjopen.bmj.com/site/about/guidelines.xhtml

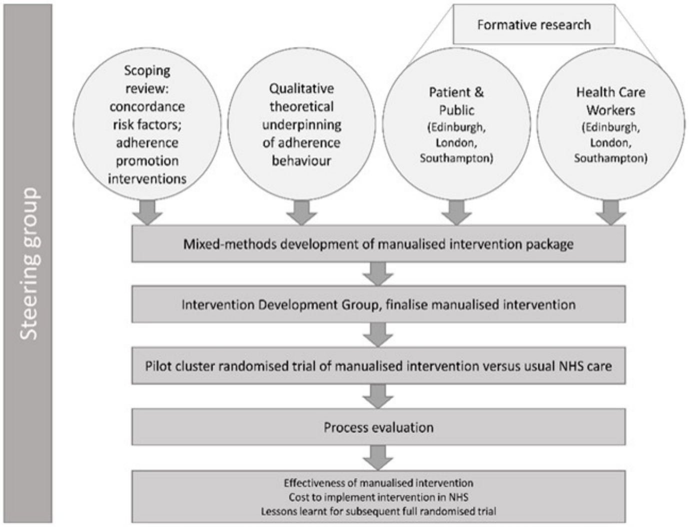

Figure 1- The IMPACT study
The six mixed-methods elements of the IMPACT [Intervening with a Manualised Package to Achieve treatment adherence in people with Tuberculosis] study and the relationships between them. The study is guided by a steering group, which has oversight over the entire process and culminates in a pilot study with associated evaluatory elements.

159x92mm (150 x 150 DPI)

SPIRIT

STANDARD PROTOCOL ITEMS: RECOMMENDATIONS FOR INTERVENTIONAL TRIALS

SPIRIT 2013 Checklist: Recommended items to address in a clinical trial protocol and related documents*

Section/item	Item No	Description	Page
**Administrative information**			
Title	1	Descriptive title identifying the study design, population, interventions, and, if applicable, trial acronym	1
Trial registration	2a	Trial identifier and registry name. If not yet registered, name of intended registry	2
	2b	All items from the World Health Organization Trial Registration Data Set	1-11
Protocol version	3	Date and version identifier	5
Funding	4	Sources and types of financial, material, and other support	14
Roles and responsibilities	5a	Names, affiliations, and roles of protocol contributors	1, 14
	5b	Name and contact information for the trial sponsor	12

For peer review only

	5c	Role of study sponsor and funders, if any, in study design; collection, management, analysis, and interpretation of data; writing of the report; and the decision to submit the report for publication, including whether they will have ultimate authority over any of these activities<br><br>The key roles and responsibilities are listed in the full protocol, available online and referenced at the end of the paper https://www.fundingawards.nihr.ac.uk/award/16/88/06<br><br>(The page number indicated refers to the location in the manuscript of the link to the full protocol)	14
	5d	Composition, roles, and responsibilities of the coordinating centre, steering committee, endpoint adjudication committee, data management team, and other individuals or groups overseeing the trial, if applicable (see Item 21a for data monitoring committee)<br><br>The management of the study is described in the full protocol.<br><br>An Intervention Development Group overseeing the development of the content for the manualised intervention and advising on the design of the pilot study is also described in the full protocol.<br><br>(The page number indicated refers to the location in the manuscript of the link to the full protocol)	14
**Introduction**			
Background and rationale	6a	Description of research question and justification for undertaking the trial, including summary of relevant studies (published and unpublished) examining benefits and harms for each intervention	4
	6b	Explanation for choice of comparators	5
Objectives	7	Specific objectives or hypotheses	4-5

For peer review only

Trial design	8	Description of trial design including type of trial (eg, parallel group, crossover, factorial, single group), allocation ratio, and framework (eg, superiority, equivalence, noninferiority, exploratory)	8

Methods: Participants, interventions, and outcomes

Study setting	9	Description of study settings (eg, community clinic, academic hospital) and list of countries where data will be collected. Reference to where list of study sites can be obtained	8
Eligibility criteria	10	Inclusion and exclusion criteria for participants. If applicable, eligibility criteria for study centres and individuals who will perform the interventions (eg, surgeons, psychotherapists)	8
Interventions	11a	Interventions for each group with sufficient detail to allow replication, including how and when they will be administered	8
	11b	Criteria for discontinuing or modifying allocated interventions for a given trial participant (eg, drug dose change in response to harms, participant request, or improving/worsening disease)	8/9
		The withdrawal procedure is outlined in detail in the main protocol.	
		(The second page number indicated refers to the location in the manuscript of the link to the full protocol).	14
	11c	Strategies to improve adherence to intervention protocols, and any procedures for monitoring adherence (eg, drug tablet return, laboratory tests)	9
	11d	Relevant concomitant care and interventions that are permitted or prohibited during the trial	8/9

For peer review only - http://bmjopen.bmj.com/site/about/guidelines.xhtml

Outcomes	12	Primary, secondary, and other outcomes, including the specific measurement variable (eg, systolic blood pressure), analysis metric (eg, change from baseline, final value, time to event), method of aggregation (eg, median, proportion), and time point for each outcome. Explanation of the clinical relevance of chosen efficacy and harm outcomes is strongly recommended	9
Participant timeline	13	Time schedule of enrolment, interventions (including any run-ins and washouts), assessments, and visits for participants. A schematic diagram is highly recommended (see Figure)	9-10
Sample size	14	Estimated number of participants needed to achieve study objectives and how it was determined, including clinical and statistical assumptions supporting any sample size calculations	10-11
Recruitment	15	Strategies for achieving adequate participant enrolment to reach target sample size	8-9
**Methods: Assignment of interventions (for controlled trials)**			
Allocation:			
Sequence generation	16a	Method of generating the allocation sequence (eg, computer-generated random numbers), and list of any factors for stratification. To reduce predictability of a random sequence, details of any planned restriction (eg, blocking) should be provided in a separate document that is unavailable to those who enrol participants or assign interventions	8
		The randomisation procedure is described in the full protocol.	14
		(The second page number indicated refers to the location in the manuscript of the link to the full protocol).	

Allocation concealment mechanism	16b	Mechanism of implementing the allocation sequence (eg, central telephone; sequentially numbered, opaque, sealed envelopes), describing any steps to conceal the sequence until interventions are assigned<br><br>The randomisation procedure is described in the full protocol.<br><br>(The page number indicated refers to the location in the manuscript of the link to the full protocol).	14
Implementation	16c	Who will generate the allocation sequence, who will enrol participants, and who will assign participants to interventions	8
Blinding (masking)	17a	Who will be blinded after assignment to interventions (eg, trial participants, care providers, outcome assessors, data analysts), and how	8
	17b	If blinded, circumstances under which unblinding is permissible, and procedure for revealing a participant's allocated intervention during the trial	8
**Methods: Data collection, management, and analysis**			
Data collection methods	18a	Plans for assessment and collection of outcome, baseline, and other trial data, including any related processes to promote data quality (eg, duplicate measurements, training of assessors) and a description of study instruments (eg, questionnaires, laboratory tests) along with their reliability and validity, if known. Reference to where data collection forms can be found, if not in the protocol<br><br>These will all be available as appendices in the full protocol. The Case Report Form will be added to the study protocol before the pilot study begins.<br><br>(The page number indicated refers to the location in the manuscript of the link to the full protocol).	14

For peer review only - http://bmjopen.bmj.com/site/about/guidelines.xhtml

	18b	Plans to promote participant retention and complete follow-up, including list of any outcome data to be collected for participants who discontinue or deviate from intervention protocols<br><br>This is described in the full protocol.<br><br>(The page number indicated refers to the location in the manuscript of the link to the full protocol).	14
Data management	19	Plans for data entry, coding, security, and storage, including any related processes to promote data quality (eg, double data entry; range checks for data values). Reference to where details of data management procedures can be found, if not in the protocol.<br><br>Details for data management are listed in the full protocol.<br><br>(The page number indicated refers to the location in the manuscript of the link to the full protocol).	14
Statistical methods	20a	Statistical methods for analysing primary and secondary outcomes. Reference to where other details of the statistical analysis plan can be found, if not in the protocol	10
	20b	Methods for any additional analyses (eg, subgroup and adjusted analyses)	10
	20c	Definition of analysis population relating to protocol non-adherence (eg, as randomised analysis), and any statistical methods to handle missing data (eg, multiple imputation)	10

Methods: Monitoring

Data monitoring	21a	Composition of data monitoring committee (DMC); summary of its role and reporting structure; statement of whether it is independent from the sponsor and competing interests; and reference to where further details about its charter can be found, if not in the protocol. Alternatively, an explanation of why a DMC is not needed<br><br>A formal data monitoring committee is not required for this study. See 5d for Steering committee oversight.	
	21b	Description of any interim analyses and stopping guidelines, including who will have access to these interim results and make the final decision to terminate the trial<br><br>This is a pilot study with limited power to detect important changes in outcome and therefore there are no plans to terminate the trial early based on interim results.	
Harms	22	Plans for collecting, assessing, reporting, and managing solicited and spontaneously reported adverse events and other unintended effects of trial interventions or trial conduct<br><br>Adverse event reporting is described in the full protocol.<br><br>(The page number indicated refers to the location in the manuscript of the link to the full protocol).	14

Auditing	23	Frequency and procedures for auditing trial conduct, if any, and whether the process will be independent from investigators and the sponsor<br><br>Outlined in the full protocol.<br><br>(The page number indicated refers to the location in the manuscript of the link to the full protocol).	14
**Ethics and dissemination**			
Research ethics approval	24	Plans for seeking research ethics committee/institutional review board (REC/IRB) approval	12
Protocol amendments	25	Plans for communicating important protocol modifications (eg, changes to eligibility criteria, outcomes, analyses) to relevant parties (eg, investigators, REC/IRBs, trial participants, trial registries, journals, regulators)<br><br>Approval for any modification to the protocol will be sought from the sponsor, REC, and subsequently communicated to the Research Offices at all study sites.	
Consent or assent	26a	Who will obtain informed consent or assent from potential trial participants or authorised surrogates, and how (see Item 32)	
	26b	Additional consent provisions for collection and use of participant data and biological specimens in ancillary studies, if applicable	N/A

For peer review only

Confidentiality	27	How personal information about potential and enrolled participants will be collected, shared, and maintained in order to protect confidentiality before, during, and after the trial<br><br>Outlined in the full protocol.<br><br>(The page number indicated refers to the location in the manuscript of the link to the full protocol).	14
Declaration of interests	28	Financial and other competing interests for principal investigators for the overall trial and each study site	14
Access to data	29	Statement of who will have access to the final trial dataset, and disclosure of contractual agreements that limit such access for investigators	12
Ancillary and post-trial care	30	Provisions, if any, for ancillary and post-trial care, and for compensation to those who suffer harm from trial participation<br><br>Outlined in the full protocol.<br><br>(The page number indicated refers to the location in the manuscript of the link to the full protocol).	14
Dissemination policy	31a	Plans for investigators and sponsor to communicate trial results to participants, healthcare professionals, the public, and other relevant groups (eg, via publication, reporting in results databases, or other data sharing arrangements), including any publication restrictions<br><br>Outlined in the full protocol.<br><br>(The page number indicated refers to the location in the manuscript of the link to the full protocol).	14

For peer review only - http://bmjopen.bmj.com/site/about/guidelines.xhtml

	31b	Authorship eligibility guidelines and any intended use of professional writers<br><br>Academic papers will be published according to UCL's policy on publications https://www.ucl.ac.uk/library/open-access/ucl-publications-policy-2012<br><br>Professional writers will not be used.	
	31c	Plans, if any, for granting public access to the full protocol, participant-level dataset, and statistical code<br><br>The full protocol is available on the NIHR website https://fundingawards.nihr.ac.uk/award/16/88/06. All research outputs will be deposited in the UCL repository in Open Access. We have no plans for the dataset to be made available.	
**Appendices**			
Informed consent materials	32	Model consent form and other related documentation given to participants and authorised surrogates<br><br>Model consent form and PIS will be made available on the study website.	
Biological specimens	33	Plans for collection, laboratory evaluation, and storage of biological specimens for genetic or molecular analysis in the current trial and for future use in ancillary studies, if applicable	N/A

*It is strongly recommended that this checklist be read in conjunction with the SPIRIT 2013 Explanation & Elaboration for important clarification on the items. Amendments to the protocol should be tracked and dated. The SPIRIT checklist is copyrighted by the SPIRIT Group under the Creative Commons "Attribution-NonCommercial-NoDerivs 3.0 Unported" license.

For peer review only
